# Trends and levels of the global, regional, and national burden of pulmonary arterial hypertension from 1990 to 2021: findings from the global burden of disease study 2021

**DOI:** 10.3389/fmed.2024.1515961

**Published:** 2024-12-10

**Authors:** Le Liu, Chen Li, Jing Cai, Renjing Kong, Yanjiao Wang, Yi Wang, Shuang Li, Junkun Zhan, Youshuo Liu

**Affiliations:** ^1^Department of Geriatrics, The Second Xiangya Hospital, Central South University, Changsha, Hunan, China; ^2^Institute of Aging and Age-related Disease Research, Central South University, Changsha, Hunan, China

**Keywords:** disease burden, pulmonary arterial hypertension, epidemiology, prevalence, sociodemographic index

## Abstract

**Background:**

Pulmonary arterial hypertension (PAH) is a severe and progressive lung disease that significantly impairs patients’ health and imposes heavy clinical and economic burdens. Currently, there is a lack of comprehensive epidemiological analysis on the global burden and trends of PAH.

**Methods:**

We estimated the prevalence, mortality, disability-adjusted life years (DALYs) of PAH from 1990 to 2021 using the results of the Global Burden of Diseases, Injuries, and Risk Factors Study (GBD). The average annual percentage changes were used to estimate the trends of PAH across 21 regions and 204 countries and territories.

**Results:**

From 1990 to 2021, the number of prevalent cases and deaths associated with PAH worldwide increased by 81.5 and 48.4%. However, the age-standardized prevalence rate of PAH remained relatively stable, while the age-standardized mortality rate and DALYs declined. In 2021, the global age-standardized prevalence rate of PAH was 2.28 per 100,000, with 1.78 per 100,000 in males and 2.75 per 100,000 in females. The age-standardized mortality rate of PAH globally was 0.27 per 100,000, and the age-standardized DALYs was 8.24 per 100,000. Among the 21 regions, Western Europe had the highest age-standardized prevalence rate (3.56 per 100,000), while North Africa and the Middle East had the highest age-standardized mortality rate (0.44 per 100,000) and DALYs (14.81 per 100,000). Additionally, older individuals and females are at higher risk of PAH. The age-standardized mortality rate and DALYs associated with PAH increase with age, peaking in the 95+ age group. As the sociodemographic index increased, the age-standardized prevalence rates showed an upward trend, while both the age-standardized mortality rates and DALYs exhibited a downward trend.

**Conclusion:**

From 1990 to 2021, the overall trend of PAH burden presents regional and national variations and differs by age, sex, and sociodemographic index. These findings emphasize the importance of implementing targeted interventions to alleviate the burden of PAH.

## 1 Introduction

Pulmonary arterial hypertension (PAH) is a severe, progressive lung disease manifested by pulmonary vasculopathy, elevated pulmonary artery pressure (1), and deteriorated right ventricular function (2), leading to progressive dyspnea, exercise limitation, right ventricular failure, and ultimately premature death (3). The symptoms of PAH are relatively non-specific and the onset is often insidious. Dyspnea is the first symptom in 60% of PAH patients, followed by fatigue, presyncope, syncope, and chest pain. In the later stages of the disease, these symptoms may occur with minimal exertion or even at rest (4).

In the United States, the estimated prevalence of PAH is 1.06 per 100,000 (5), while a French study reported a prevalence of 1.50 per 100,000 (6). As a life-threatening disease, before the development of specific treatments for PAH, the 5-year survival rate after diagnosis was 34% (7). In recent years, with the development and application of specific drugs for PAH, the 5-year survival rate of patients with PAH has significantly improved (a study in 2015 showed an increase to over 60%) (5, 8). Currently, there is a significant lack of global epidemiological research on PAH. It is essential to gather the latest estimated data worldwide to gain a deeper understanding of the disease burden and its trends. Conducting global epidemiological studies on PAH enables policymakers to understand the situation and formulate effective prevention and control strategies. Precise PAH prevention, screening, and treatment strategies can be tailored based on various factors such as region, country, sociodemographic indices, age, and gender, ultimately aiming to improve patient outcomes.

This study reports the burden of PAH across 204 countries and territories from 1990 to 2021, based on the latest estimates of prevalence, mortality, and disability-adjusted life years (DALYs) from the Global Burden of Diseases, Injuries, and Risk Factors Study (GBD) 2021, to provide insights into tailored policies and strategies for the prevention, screening, and treatment of PAH, ultimately benefiting patients with this condition.

## 2 Methods

### 2.1 Data sources

The data for this study comes from GBD 2021^1^ (9, 10). GBD 2021 is a comprehensive study of global health loss. GBD 2021 provides the latest information on the distribution and burden of diseases and injuries across different times, ages, genders, locations, and sociodemographic groups. It encompasses 100,983 data sources and estimates for 204 countries and territories, categorized into 21 regions and 7 super-regions, evaluating the burden of 371 diseases and injuries in total. The data comes from vital registration systems, verbal autopsies, censuses, household surveys, disease-specific registries, health service utilization data, and other sources (9). We obtained data on the prevalence, mortality, and DALYs of PAH, stratified by age, gender, region, country, and sociodemographic index (SDI) categories. GBD 2021 could provide each estimate along with its corresponding uncertainty interval (UI). Spatiotemporal Gaussian Process Regression (ST-GPR) modeling was utilized to enable smoothing across age, time, and location in areas where comprehensive datasets were absent. Additionally, the Bayesian noise reduction algorithm was implemented to address the challenges posed by zero counts and low numbers for rare causes. The data processing involves adjustments for heterogeneity and biases, coupled with rigorous uncertainty analysis through Monte Carlo simulations, ensuring robust and reliable estimates.

### 2.2 Definitions of PAH

GBD 2021 defines PAH, which aligns with WHO Group 1 pulmonary hypertension, as a clinical diagnosis of pulmonary hypertension, supported by diagnostic evidence from right heart catheterization or echocardiography. If the study authors confirmed the diagnosis by reviewing the medical records of catheterization or echocardiography results, then PAH identified through International Classification of Diseases (ICD) codes would be included in the study. All other forms of pulmonary hypertension were excluded from this etiology (3, 9, 11).

### 2.3 Sociodemographic index

The SDI is a comprehensive indicator launched by the Institute for Health Metrics and Evaluation (IHME) in 2015. It is used to provide insights into the social and economic conditions that impact health outcomes in specific regions, emphasizing the interconnections between social development and population health outcomes. And it is a measure comprising lagged distributed income *per capita*, average years of education for those aged 15 or above, and Total Fertility Rate Under 25. In the GBD 2021, 204 countries and territories are classified into five SDI regions: low, low-middle, middle, high-middle, and high (9, 12, 13).

### 2.4 Regional division

This study utilized the GBD 2021 classification system to divide the world into 21 geographical regions based on epidemiological similarity and geographical proximity. This classification aids in gaining a more granular understanding of the variations in disease burden across different regions of the world, enabling the formulation of targeted public health policies and interventions.

### 2.5 Collection of indicators

We extracted data on the number of cases, prevalence, mortality, and DALYs of PAH, and generated trend graphs for the prevalence, mortality, and DALYs of PAH from 1990 to 2021. The prevalence was modeled utilizing DisMod-MR 2.1 (Disease Modeling Meta-Regression; version 2.1). Estimates of the cause of death were modeled using the Cause of Death Ensemble model (CODEm). The sum of years lived with disability (YLDs) and years of life lost (YLLs) was used to calculate DALYs.

### 2.6 Statistical analysis

To gain a deeper understanding of the temporal trends in the age-standardized rates of prevalence, mortality, and DALYs, we have employed joinpoint regression analysis, which has enabled us to estimate the average annual percentage change (AAPC) along with its 95% confidence interval (CI). We calculated the AAPC between 1990 and 2021, with the value of AAPC indicating the magnitude of the annual change (increase, decrease, or no change). If the estimated percentage of annual change is >0 (or <0), we consider the corresponding rate to be in an increasing (or decreasing) trend (14).

We compared the prevalence, mortality, and DALYs of PAH between males and females, across different age groups (0–4 years, 5–9 years, 10–14 years, 15–19 years, 20–24 years, 25–29 years, 30–34 years, 35–39 years, 40–44 years, 45–49 years, 50–54 years, 55–59 years, 60–64 years, 65–69 years, 70–74 years, 75–79 years, 80–84 years, 85–89 years, 90–94 years, and 95+ years), across different countries and regions, and among different levels of SDI.

We utilized the autoregressive integrated moving average (ARIMA) model to forecast the trends in prevalence, mortality, and DALYs of PAH from 2022 to 2050. The ARIMA model is a time series forecasting and analysis method. In ARIMA (p, d, q), AR stands for “Autoregressive,” where p represents the number of autoregressive terms. MA stands for “Moving Average,” where q represents the number of terms in the moving average. Lastly, d denotes the number of differencing operations (orders) performed to make the series stationary (15).

All statistics were performed using R program (version 4.1.3), GraphPad Prism (version 8.0) and Joinpoint Regression program (version 5.0.2).

## 3 Results

### 3.1 Global trends

In 1990, there were an estimated 105,703 (95% UI 86,381, 130,334) cases of PAH worldwide, which rose to 191,808 (155,357, 235,787) cases in 2021, marking an increase of 81.5% from 1990 to 2021. The age-standardized prevalence rate of the global population remained relatively stable, with 2.30 (1.87, 2.82) per 100,000 population in 1990 and 2.28 (1.85, 2.80) per 100,000 population in 2021. The annual average trend was −0.03% (95% confidence interval [CI] −0.05, −0.01%) (Table 1).

**Table 1:** Age-standardized prevalence rate of PAH globally and stratified by gender, age and SDI levels, 1990–2021.

	Prevalence (95% UI)				
	Cases in 1990	Age-standardized rate in 1990 (per 100,000)	Cases in 2021	Age-standardized rate in 2021 (per 100,000)	1990–2021 AAPC (95% CI)
Global	105,703 (86,381, 130,334)	2.30 (1.87, 2.82)	191,808 (155,357, 235,787)	2.28 (1.85, 2.80)	−0.03 (−0.05, −0.01)
Sex					
Female	66,282 (54,058, 81,489)	2.81 (2.29, 3.46)	118,682 (95,923, 146,291)	2.75 (2.24, 3.39)	−0.07 (−0.09, −0.05)
Male	39,421 (32,159, 48,677)	1.75 (1.42, 2.13)	73,127 (58,944, 89,629)	1.78 (1.44, 2.17)	0.06 (0.04, 0.09)
Age group (years)					
0–4	1,404 (1,003, 1903)	0.23 (0.16, 0.31)	1,523 (1,088, 2081)	0.23 (0.17, 0.32)	0.07 (0.04, 0.10)
5–9	2,675 (1930, 3,507)	0.46 (0.33, 0.60)	3,133 (2,249, 4,120)	0.46 (0.33, 0.60)	−0.02 (−0.03, −0.01)
10–14	3,536 (2,365, 4,993)	0.66 (0.44, 0.93)	4,318 (2,876, 6,154)	0.65 (0.43, 0.92)	−0.06 (−0.07, −0.05)
15–19	4,869 (2,878, 7,375)	0.94 (0.55, 1.42)	5,736 (3,366, 8,768)	0.92 (0.54, 1.41)	−0.07 (−0.09, −0.04)
20–24	6,168 (4,167, 8,920)	1.25 (0.85, 1.81)	7,380 (4,942, 10,703)	1.24 (0.83,1.79)	−0.04 (−0.06, −0.03)
25–29	7,113 (4,793, 10,347)	1.61 (1.08, 2.34)	9,345 (6,238, 13,584)	1.59 (1.06, 2.31)	−0.04 (−0.04, −0.03)
30–34	7,668 (4,967, 11,298)	1.99 (1.29, 2.93)	12,034 (7,694, 17,877)	1.99 (1.27, 2.96)	0.00 (−0.01, 0.01)
35–39	8,381 (5,814, 11,738)	2.38 (1.65, 3.33)	13,366 (9,219, 18,800)	2.38 (1.64, 3.35)	0.00 (−0.02, 0.02)
40–44	7,865 (5,401, 10,926)	2.75 (1.89, 3.81)	13,750 (9,392, 19,277)	2.75 (1.88, 3.85)	0.00 (−0.02, 0.02)
45–49	7,277 (4,716, 10,659)	3.13 (2.03, 4.59)	15,017 (9,643, 21,952)	3.17 (2.04, 4.64)	0.05 (0.01, 0.09)
50–54	8,051 (5,697, 11,415)	3.79 (2.68, 5.37)	16,909 (11,845, 23,968)	3.80 (2.66, 5.39)	0.00 (−0.04, 0.05)
55–59	8,369 (5,748, 11,794)	4.52 (3.10, 6.37)	18,013 (12,334, 25,501)	4.55 (3.12, 6.44)	0.03 (0.01, 0.06)
60–64	8,700 (5,678, 12,690)	5.42 (3.54, 7.90)	17,199 (11,303, 25,275)	5.37 (3.53, 7.90)	−0.02 (−0.04, −0.01)
65–69	7,974 (5,685, 10,966)	6.45 (4.60, 8.87)	17,268 (12,304, 23,728)	6.26 (4.46, 8.60)	−0.11 (−0.16, −0.05)
70–74	6,273 (4,469, 8,562)	7.41 (5.28, 10.11)	15,091 (10,763, 20,724)	7.33 (5.23, 10.07)	−0.03 (−0.07, 0.00)
75–79	5,207 (3,541, 7,474)	8.46 (5.75, 12.14)	10,542 (7,018, 15,278)	7.99 (5.32, 11.58)	−0.19 (−0.24, −0.14)
80–84	2,812 (1956, 3,901)	7.95 (5.53, 11.03)	6,670 (4,637, 9,265)	7.62 (5.29, 10.58)	−0.14 (−0.18, −0.10)
85–89	1,052 (699, 1,499)	6.96 (4.63, 9.92)	3,115 (2055, 4,471)	6.81 (4.49, 9.78)	−0.07 (−0.09, −0.04)
90–94	257 (164, 386)	6.00 (3.84, 9.01)	1,090 (687, 1,648)	6.09 (3.84, 9.21)	0.03 (−0.05, 0.11)
95+	53 (31, 86)	5.21 (3.08, 8.46)	312 (184, 506)	5.73 (3.37, 9.27)	0.31 (0.21, 0.40)
SDI level					
High	27,054 (21,977, 33,298)	2.67 (2.17, 3.27)	41,452 (33,445, 51,588)	2.64 (2.15, 3.23)	−0.04 (−0.06, −0.01)
High-middle	27,439 (22,296, 33,800)	2.61 (2.12, 3.21)	42,636 (34,236, 52,932)	2.54 (2.07, 3.12)	−0.10 (−0.13, −0.06)
Middle	29,051 (23,769, 35,769)	2.07 (1.68, 2.54)	59,667 (48,065, 73,648)	2.21 (1.80, 2.71)	0.21 (0.19, 0.24)
Low-middle	15,079 (12,417, 18,609)	1.77 (1.43, 2.15)	32,715 (26,546, 40,620)	1.90 (1.53, 2.33)	0.23 (0.22, 0.24)
Low	6,966 (5,737, 8,609)	2.08 (1.68, 2.53)	15,181 (12,474, 18,747)	1.94 (1.58, 2.36)	−0.21 (−0.24, −0.19)

AAPC, average annual percentage change; CI, confidence interval; SDI, sociodemographic index; UI, uncertainty interval.

There were an estimated 14,842 (12,370, 17,485) deaths in 1990 and 22,021 (18,239, 25,352) deaths in 2021 from PAH worldwide, marking an increase of 48.4%. The age-standardized mortality rate declined from 0.35 (0.29, 0.42) per 100,000 population in 1990 to 0.27 (0.23, 0.32) per 100,000 population in 2021, with an average annual decline of −0.82% (−0.95, −0.68%) (Table 2).

**Table 2:** Age-standardized mortality rate of PAH globally and stratified by gender, age and SDI levels, 1990–2021.

	Mortality (95% UI)				
	Cases in 1990	Age-standardized rate in 1990 (per 100,000)	Cases in 2021	Age-standardized rate in 2021 (per 100,000)	1990–2021 AAPC (95% CI)
Global	14,842 (12,370, 17,485)	0.35 (0.29, 0.42)	22,021 (18,239, 25,352)	0.27 (0.23, 0.32)	−0.82 (−0.95, −0.68)
Sex					
Female	7,516 (5,058, 10,161)	0.34 (0.23, 0.45)	12,442 (10,001, 15,376)	0.28 (0.22, 0.34)	−0.61 (−0.73, −0.49)
Male	7,326 (5,917, 8,715)	0.37 (0.29, 0.46)	9,579 (7,516, 11,703)	0.27 (0.21, 0.33)	−1.07 (−1.27, −0.87)
Age group (years)					
0–4	3,616 (2037, 5,150)	0.58 (0.33, 0.83)	1,409 (1,079, 1,785)	0.21 (0.16, 0.27)	−3.16 (−3.37, −2.94)
5–9	245 (176, 323)	0.04 (0.03, 0.06)	151 (121, 188)	0.02 (0.02, 0.03)	−2.11 (−2.33, −1.89)
10–14	188 (141, 236)	0.04 (0.03, 0.04)	154 (122, 194)	0.02 (0.02, 0.03)	−1.34 (−1.58, −1.10)
15–19	298 (232, 388)	0.06 (0.04, 0.07)	258 (207, 334)	0.04 (0.03, 0.05)	−1.06 (−1.20, −0.91)
20–24	336 (255, 436)	0.07 (0.05, 0.09)	328 (259, 417)	0.05 (0.04, 0.07)	−0.72 (−0.82, −0.62)
25–29	412 (321, 517)	0.09 (0.07, 0.12)	420 (348, 507)	0.07 (0.06, 0.09)	−0.90 (−0.98, −0.82)
30–34	349 (270,439)	0.09 (0.07, 0.11)	400 (335,478)	0.07 (0.06, 0.08)	−1.03 (−1.21, −0.85)
35–39	428 (337, 532)	0.12 (0.10, 0.15)	479 (408, 560)	0.09 (0.07, 0.10)	−1.14 (−1.28, −0.99)
40–44	508 (408, 641)	0.18 (0.14, 0.22)	619 (525, 728)	0.12 (0.10, 0.15)	−1.17 (−1.37, −0.97)
45–49	378 (300, 471)	0.16 (0.13, 0.20)	520 (447, 618)	0.11 (0.09, 0.13)	−1.26 (−1.41, −1.10)
50–54	542 (432, 676)	0.25 (0.20, 0.32)	736 (622, 862)	0.17 (0.14, 0.19)	−1.40 (−1.57, −1.23)
55–59	730 (580, 906)	0.39 (0.31, 0.49)	1,102 (860, 1,317)	0.28 (0.22, 0.33)	−1.16 (−1.36, −0.97)
60–64	834 (664, 1,032)	0.52 (0.41, 0.64)	1,148 (912, 1,341)	0.36 (0.28, 0.42)	−1.17 (−1.31, −1.04)
65–69	1,059 (839, 1,315)	0.86 (0.68, 1.06)	1751 (1,383, 2081)	0.63 (0.50, 0.75)	−0.95 (−1.08, −0.81)
70–74	1,162 (904, 1,458)	1.37 (1.07, 1.72)	2,233 (1,686, 2,660)	1.08 (0.82, 1.29)	−0.76 (−0.93, −0.59)
75–79	1,367 (1,073, 1,669)	2.22 (1.74, 2.71)	2,547 (1884, 3,075)	1.93 (1.43, 2.33)	−0.46 (−0.73, −0.20)
80–84	1,171 (893, 1,484)	3.31 (2.53, 4.19)	2,794 (2065, 3,388)	3.19 (2.36, 3.87)	−0.10 (−0.34, 0.13)
85–89	792 (603, 994)	5.24 (3.99, 6.58)	2,617 (2027, 3,071)	5.72 (4.43, 6.72)	0.26 (0.01, 0.50)
90–94	328 (251, 399)	7.66 (5.87,9.30)	1,634 (1,270, 1,922)	9.13 (7.1, 10.74)	0.60 (0.40, 0.80)
95+	101 (77, 123)	9.96 (7.58, 12.06)	719 (518, 850)	13.19 (9.51, 15.60)	0.91 (0.78, 1.05)
SDI level					
High	2,617 (2,379, 2,850)	0.26 (0.24, 0.28)	4,621 (3,919, 5,054)	0.22 (0.19, 0.23)	−0.59 (−0.75, −0.44)
High-middle	3,214 (2,772, 3,908)	0.35 (0.31, 0.43)	4,326 (3,594, 5,141)	0.24 (0.20, 0.29)	−1.22 (−1.50, −0.95)
Middle	4,729 (3,774, 5,852)	0.45 (0.35, 0.58)	7,548 (5,141, 9,026)	0.33 (0.22, 0.39)	−1.10 (−1.31, −0.88)
Low-middle	3,125 (2,220, 3,904)	0.35 (0.22, 0.47)	3,728 (2,757, 5,091)	0.26 (0.18, 0.38)	−0.89 (−1.03, −0.75)
Low	1,145 (740, 1,755)	0.32 (0.15, 0.51)	1782 (1,147, 2,544)	0.27 (0.15, 0.40)	−0.53 (−0.81, −0.25)

AAPC, average annual percentage change; CI, confidence interval; SDI, sociodemographic index; UI, uncertainty interval.

From 1990 to 2021, there was a notable downward trend in DALYs associated with PAH. The age-standardized DALYs declined from 13.21 (10.78, 15.36) per 100,000 population in 1990 to 8.24 (7.14, 9.39) per 100,000 population in 2021, with an average annual trend of −1.52% (−1.64, −1.40%) (Table 3).

**Table 3:** Age-standardized DALYs of PAH globally and stratified by gender, age and SDI levels, 1990–2021.

	DALYs (95% UI)				
	Cases in 1990	Age-standardized rate in 1990 (per 100,000)	Cases in 2021	Age-standardized rate in 2021 (per 100,000)	1990–2021 AAPC (95% CI)
Global	687,419 (535,241, 813,086)	13.21 (10.78, 15.36)	642,104 (552,273, 728,993)	8.24 (7.14, 9.39)	−1.52 (−1.64, −1.40)
Sex					
Female	317,734 (194,732, 444,009)	12.32 (7.78, 16.80)	342,468 (282,652, 430,645)	8.39 (6.92, 10.53)	−1.26 (−1.37, −1.15)
Male	369,686 (316,035, 424,978)	14.09 (11.89, 16.35)	299,636 (246,726, 348,331)	8.06 (6.72, 9.36)	−1.80 (−1.90, −1.70)
Age group (years)					
0–4	323,267 (182,383, 459,664)	52.15 (29.42, 74.15)	125,957 (96,511, 159,398)	19.14 (14.66, 24.22)	−3.16 (−3.37, −2.95)
5–9	20,588 (14,826, 27,101)	3.53 (2.54, 4.64)	12,813 (10,348, 15,867)	1.86 (1.51, 2.31)	−2.08 (−2.30, −1.86)
10–14	14,885 (11,380, 18,570)	2.78 (2.12, 3.47)	12,329 (9,823, 15,345)	1.85 (1.47, 2.30)	−1.31 (−1.54, −1.07)
15–19	22,070 (17,290, 28,512)	4.25 (3.33, 5.49)	19,273 (15,486, 24,753)	3.09 (2.48, 3.97)	−1.03 (−1.17, −0.89)
20–24	23,287 (17,875, 30,044)	4.73 (3.63, 6.11)	22,866 (18,252, 28,906)	3.83 (3.06, 4.84)	−0.71 (−0.80, −0.61)
25–29	26,522 (20,742, 33,058)	5.99 (4.69, 7.47)	27,206 (22,503, 32,558)	4.62 (3.82, 5.53)	−0.88 (−0.95, −0.80)
30–34	20,851 (16,289, 26,110)	5.41 (4.23, 6.77)	24,182 (20,197, 28,862)	4.00 (3.34, 4.77)	−0.99 (−1.16, −0.81)
35–39	23,342 (18,568, 28,831)	6.63 (5.27, 8.18)	26,531 (22,781, 30,793)	4.73 (4.06, 5.49)	−1.09 (−1.23, −0.95)
40–44	25,030 (20,201, 31,451)	8.74 (7.05, 10.98)	30,888 (26,220, 35,879)	6.17 (5.24, 7.17)	−1.13 (−1.32, −0.94)
45–49	16,906 (13,562, 20,774)	7.28 (5.84, 8.95)	23,738 (20,370, 27,851)	5.01 (4.30, 5.88)	−1.20 (−1.34, −1.05)
50–54	21,422 (17,252, 26,564)	10.08 (8.12, 12.50)	29,664 (25,331, 34,265)	6.67 (5.69, 7.70)	−1.34 (−1.52, −1.15)
55–59	25,218 (20,152, 31,039)	13.62 (10.88, 16.76)	38,595 (30,612, 46,131)	9.75 (7.74, 11.66)	−1.12 (−1.31, −0.92)
60–64	24,869 (19,904, 30,697)	15.48 (12.39, 19.11)	34,711 (27,817, 39,952)	10.85 (8.69, 12.48)	−1.13 (−1.26, −1.01)
65–69	26,468 (21,139, 32,626)	21.41 (17.10, 26.39)	44,135 (34,809, 52,091)	16.00 (12.62, 18.88)	−0.92 (−1.03, −0.81)
70–74	23,809 (18,632, 29,689)	28.12 (22.01, 35.07)	46,041 (34,966, 54,861)	22.37 (16.99, 26.65)	−0.75 (−0.90, −0.59)
75–79	22,320 (17,645, 27,225)	36.26 (28.66, 44.23)	41,584 (31,083, 49,954)	31.53 (23.57, 37.88)	−0.46 (−0.71, −0.21)
80–84	14,910 (11,394, 18,784)	42.15 (32.21, 53.10)	35,420 (26,374, 42,818)	40.44 (30.11, 48.89)	−0.13 (−0.35, 0.08)
85–89	7,968 (6,100, 10,012)	52.73 (40.37, 66.26)	26,172 (20,296, 30,619)	57.24 (44.39, 66.97)	0.24 (−0.01, 0.49)
90–94	2,854 (2,190, 3,460)	66.60 (51.12, 80.75)	14,181 (11,025, 16,653)	79.27 (61.63, 93.09)	0.60 (0.40, 0.79)
95+	829 (633, 1,002)	81.46 (62.20, 98.38)	5,819 (4,200, 6,869)	106.77 (77.07, 126.03)	0.88 (0.75, 1.01)
SDI level					
High	81,792 (77,185, 88,110)	9.16 (8.71, 9.90)	93,182 (84,873, 99,192)	6.16 (5.76, 6.49)	−1.29 (−1.45, −1.14)
High-middle	127,638 (106,711, 154,438)	13.14 (10.91, 16.04)	99,448 (85,757, 117,639)	6.48 (5.61, 7.87)	−2.26 (−2.49, −2.04)
Middle	210,946 (172,857, 258,349)	14.29 (11.66, 17.69)	197,171 (148,781, 232,321)	8.23 (6.26, 9.70)	−1.82 (−2.05, −1.59)
Low-middle	195,281 (117,185, 245,591)	14.92 (10.74, 18.41)	156,400 (122,426, 194,166)	9.07 (7.05, 11.60)	−1.59 (−1.70, −1.47)
Low	71,125 (48,628, 111,614)	12.42 (7.78, 19.19)	95,342 (67,471, 133,050)	9.30 (6.08, 13.20)	−0.90 (−1.02, −0.78)

AAPC, average annual percentage change; CI, confidence interval; SDI, sociodemographic index; UI, uncertainty interval; DALYs, disability-adjusted life years.

### 3.2 Global trends by sex

The age-standardized prevalence rate of PAH among females is higher than that among males. In 1990, the global age-standardized prevalence rate of PAH among females was 2.81 (2.29, 3.46) per 100,000 population, while the age-standardized prevalence rate among males was 1.75 (1.42, 2.13) per 100,000 population. By 2021, the global age-standardized prevalence rate of PAH among females was 2.75 (2.24, 3.39) per 100,000 population, while the age-standardized prevalence rate among males was 1.78 (1.44, 2.17) per 100,000 population (Table 1).

From 1990 to 2021, the global age-standardized mortality rates of PAH declined for both men and women (men: from 0.37 [0.29, 0.46] per 100,000 to 0.27 [0.21, 0.33] per 100,000; women: from 0.34 [0.23, 0.45] per 100,000 to 0.28 [0.22, 0.34] per 100,000), with a more significant decrease observed in men than in women (AAPC: −1.07% [−1.27, −0.87%] vs. -0.61% [−0.73, −0.49%]) (Table 2).

From 1990 to 2021, the decline in global age-standardized DALYs due to PAH was more pronounced among men compared to women (AAPC −1.80% [−1.90, −1.70%] vs. −1.26% [−1.37, −1.15%]). In 1990, the age-standardized DALYs per 100,000 population for men were higher than those for women (men: 14.09 [11.89, 16.35]; women: 12.32 [7.78, 16.80]). But by 2021, the age-standardized DALYs per 100,000 population for men were lower than those for women (men: 8.06 [6.72, 9.36]; women: 8.39 [6.92, 10.53]) (Table 3).

### 3.3 Global trends by age subgroup

**Figure 1:**
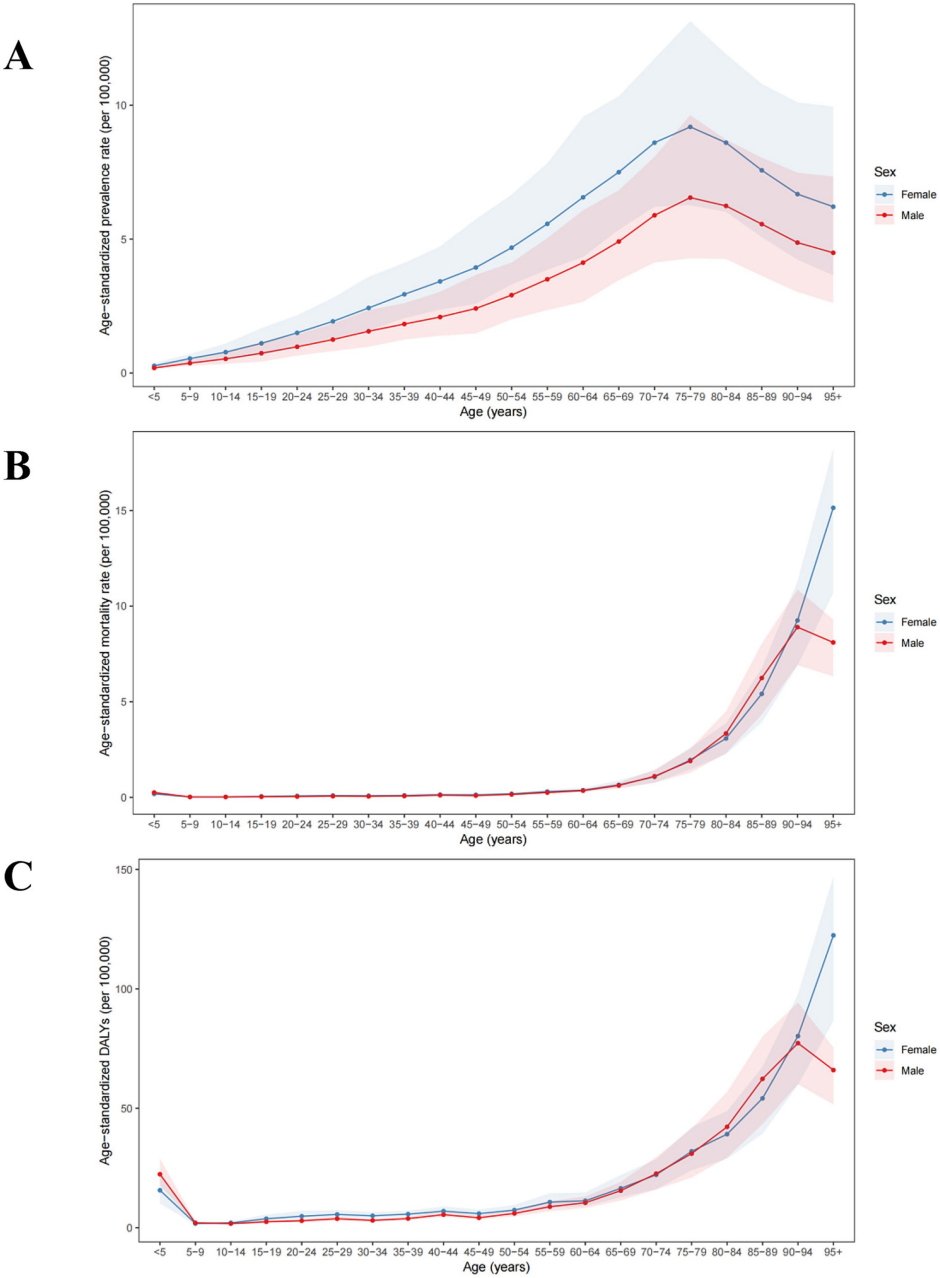
Global age-standardized **(A)** prevalence, **(B)** mortality, and **(C)** DALYs of PAH in 2021 by age group and sex, per 100,000. DALYs, disability-adjusted life years.

**Figure 2:**
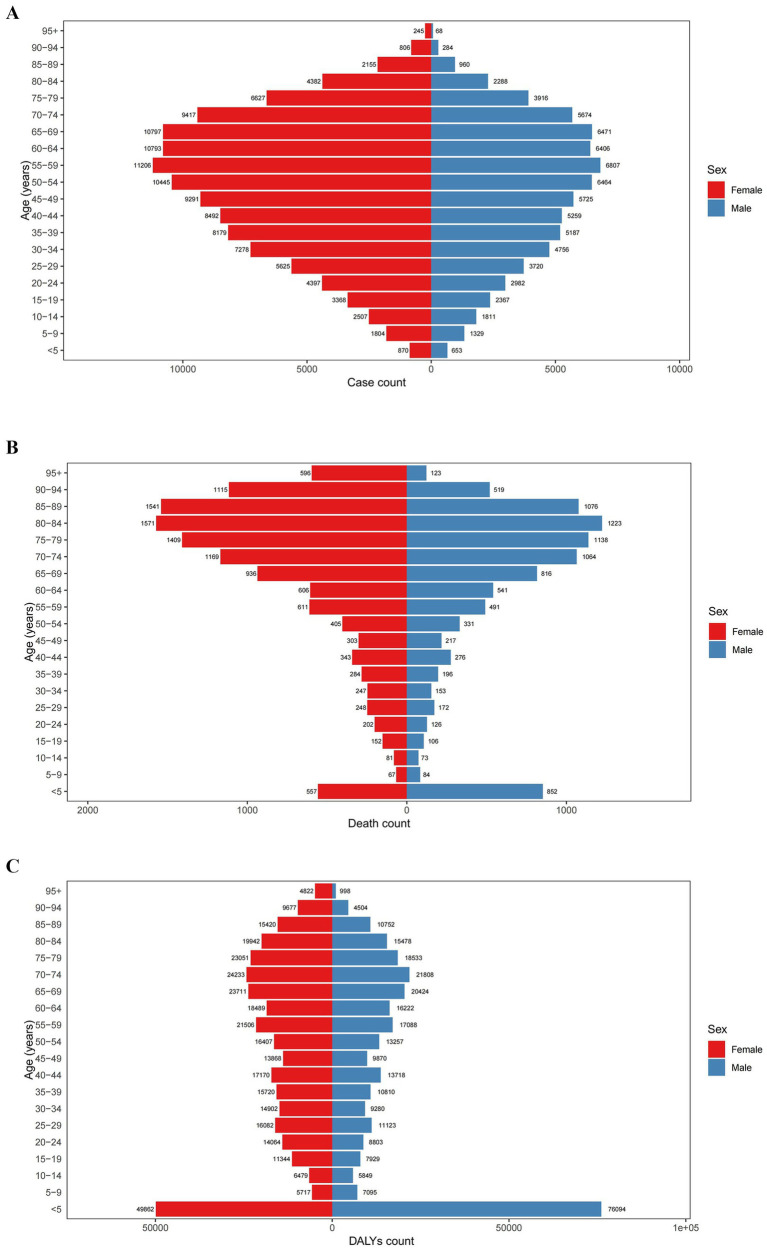
Distribution of global **(A)** prevalence, **(B)** mortality, and **(C)** DALYs of PAH in 2021 by age group and sex. DALYs, disability-adjusted life years.

The 2021 data indicate that the age-standardized prevalence rate of PAH increases with age, peaking around 80 years old before declining. No gender-specific differences were observed when stratified by sex. From 1990 to 2021, the most significant increase in age-standardized prevalence rate of PAH was observed in population aged 95+ years (from 5.21 [3.08, 8.46] to 5.73 [3.37, 9.27] per 100,000, AAPC 0.31% [0.21, 0.40%]), while the most significant decrease was seen in those aged 75–79 years (from 8.46 [5.75, 12.14] to 7.99 [5.32, 11.58] per 100,000, AAPC −0.19% [−0.24, −0.14%]) (Figures 1, 2 and Table 1).

The 2021 data reveal that the age-standardized mortality rate for PAH was lowest among those aged 5–14 years (0.02 [0.02, 0.03] per 100,000 in 2021), and thereafter, the age-standardized mortality rate increased with age, peaking among individuals aged 95+ years (13.19 [9.51, 15.60] per 100,000 in 2021). When stratified by sex, males had the highest age-standardized mortality rate in the 90–94 years age group, while females had the highest rate in the 95+ years age group. From 1990 to 2021, the age-standardized mortality rate for PAH showed a declining trend among those aged 0–84 years, while an increasing trend was observed among those aged 85+ years (Figures 1, 2 and Table 2).

The age-standardized DALYs for PAH were lowest among those aged 10–14 years (1.85 [1.47, 2.30] per 100,000), and thereafter, the age-standardized DALYs increased with age, peaking among patients aged 95 years and above (106.77 [77.07, 126.03] per 100,000) in 2021. When stratified by sex, males had the highest age-standardized DALYs in the 90–94 years age group, while females had the highest in the 95+ age group. From 1990 to 2021, the age-standardized DALYs for PAH showed a declining trend among those aged 0–84 years, whereas an increasing trend was observed among those aged 85+ years (Figures 1, 2 and Table 3).

### 3.4 Global trends by sociodemographic index

**Figure 3:**
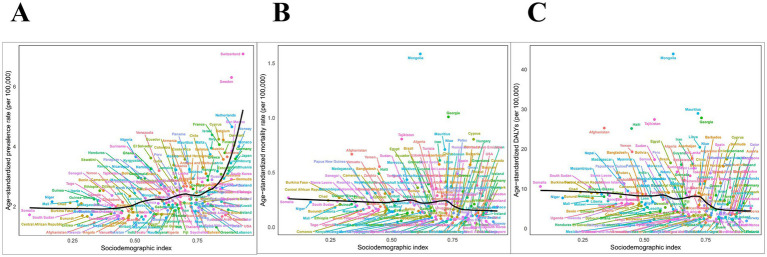
Age-standardized **(A)** prevalence, **(B)** mortality, and **(C)** DALYs of PAH from 204 countries and territories based on sociodemographic index in 2021, per 100,000. DALYs, disability-adjusted life years.

In 2021, the age-standardized prevalence rates were highest in high SDI regions and lowest in low-middle SDI regions. The age-standardized mortality rates were highest in middle SDI regions and lowest in high SDI regions. Conversely, the age-standardized DALYs were highest in low SDI regions and lowest in high SDI regions. As SDI increased, the age-standardized prevalence rate showed an upward trend, while both the age-standardized mortality rates and the age-standardized DALYs exhibited a downward trend (Figure 3 and Tables 1–3).

**Figure 4:**
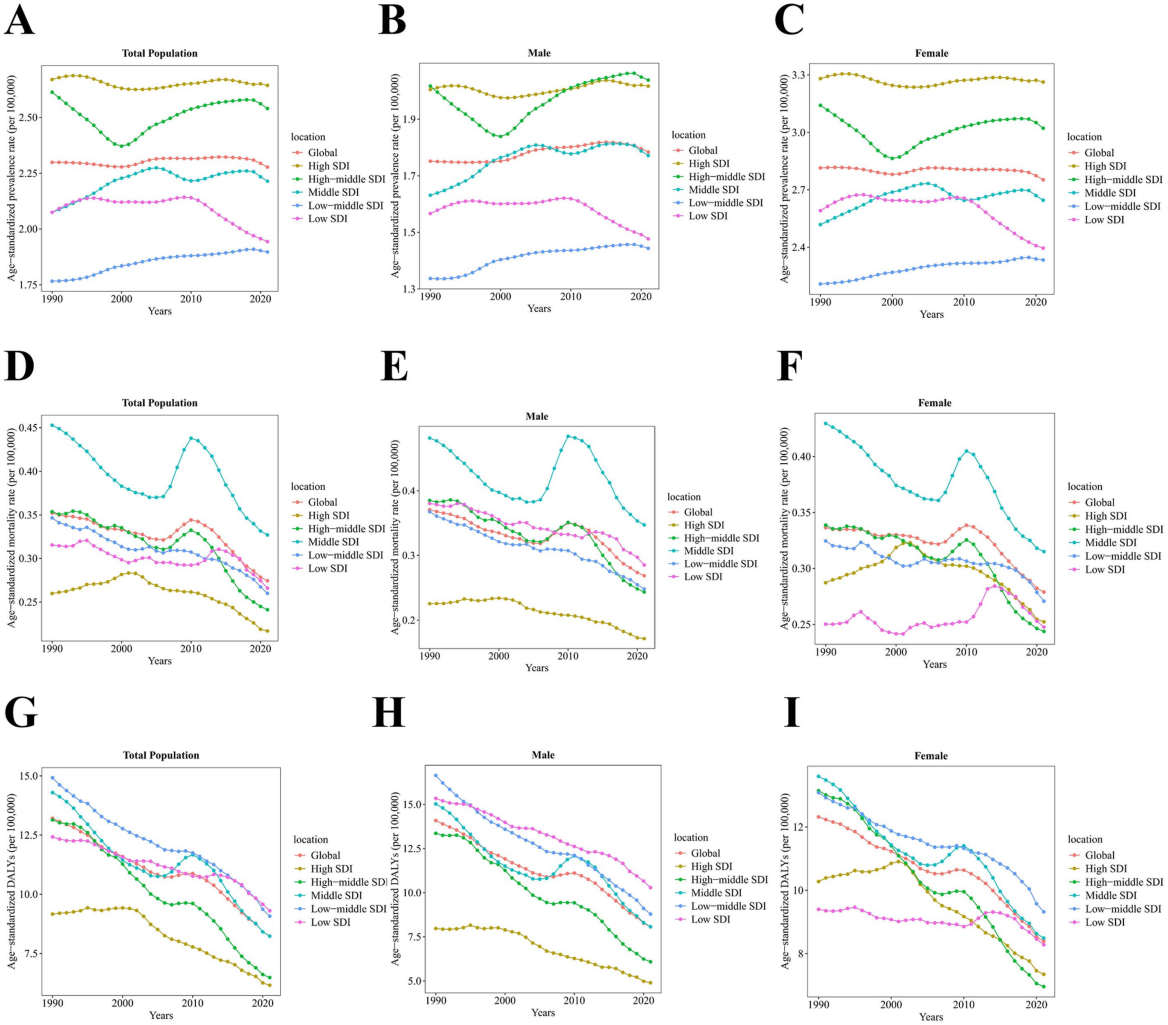
Temporal trend of age-standardized prevalence, mortality, and DALYs of PAH from 1990 to 2021 at global and sociodemographic index levels by sex, per 100,000. The temporal trend of age-standardized prevalence rate among **(A)** total population, **(B)** males, and **(C)** females. The temporal trend of age-standardized mortality rate among **(D)** total population, **(E)** males, and **(F)** females. The temporal trend of age-standardized DALYs among **(G)** total population, **(H)** males, and **(I)** females. DALYs, disability-adjusted life years. SDI, sociodemographic index.

Between 1990 and 2021, the age-standardized prevalence rate increased the most significantly in low-middle SDI regions (AAPC 0.23% [0.22, 0.24%]), whereas it decreased the most in low SDI regions (AAPC −0.21% [−0.24, −0.19%]). From 1990 to 2021, both the age-standardized mortality rate and the age-standardized DALYs exhibited a declining trend across all SDI regions, with the most substantial reductions observed in high-middle SDI regions (age-standardized mortality rate: AAPC −1.22% [−1.50, −0.95%], age-standardized DALYs: AAPC −2.26% [−2.49, −2.04%]) (Figure 4 and Tables 1–3).

**Figure 5:**
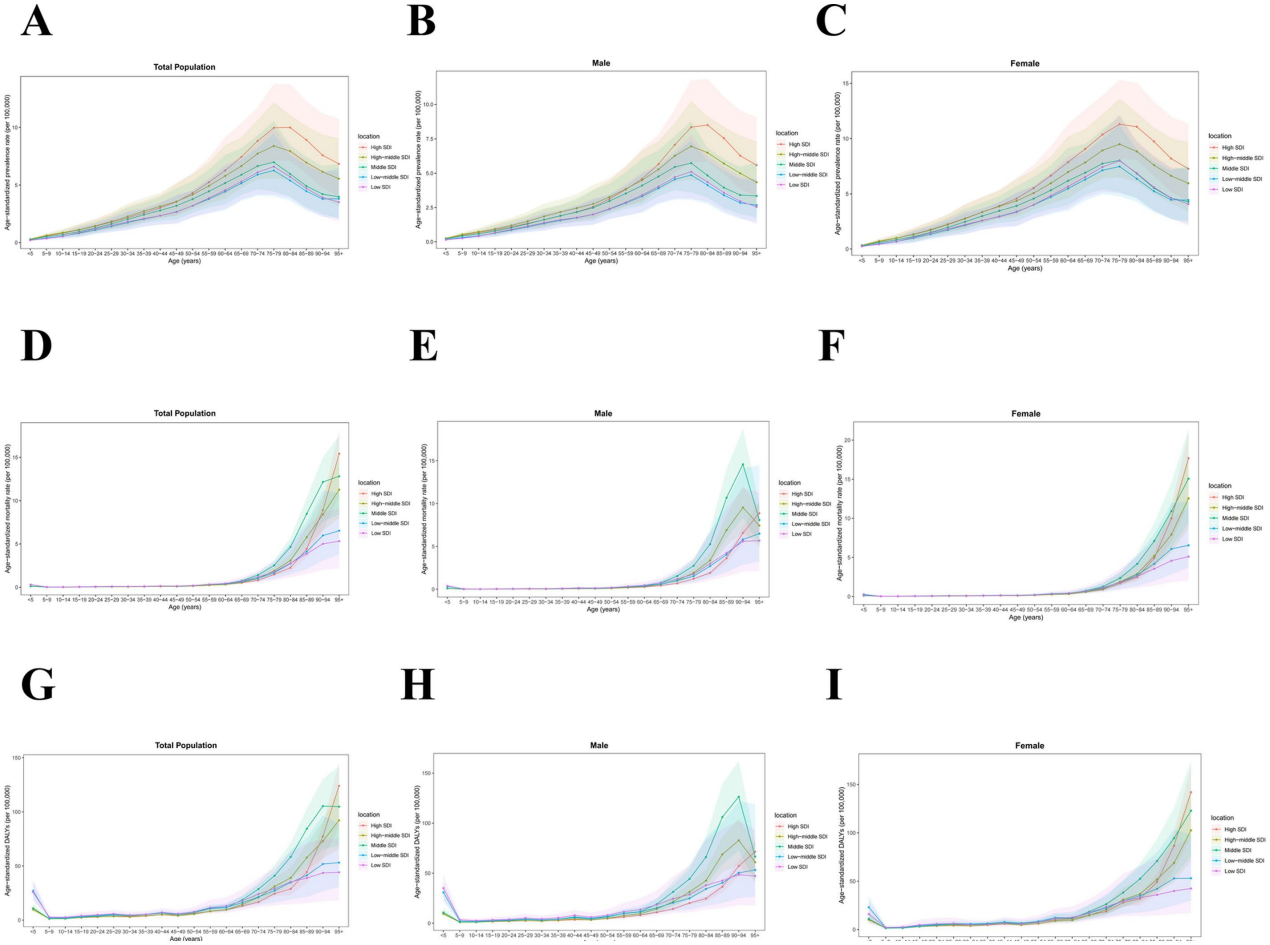
Age-standardized prevalence, mortality, and DALYs of PAH in 2021 at sociodemographic index levels by age group and sex, per 100,000. The age-standardized prevalence rate among **(A)** total population, **(B)** males, and **(C)** females. The age-standardized mortality rate among **(D)** total population, **(E)** males, and **(F)** females. The age-standardized DALYs among **(G)** total population, **(H)** males, and **(I)** females. DALYs, disability-adjusted life years. SDI, sociodemographic index.

The age-standardized prevalence rates for PAH in all five SDI regions increased with age up to 79 years, then declined after 80 years of age. The age-standardized mortality rates for PAH in these five SDI regions all increased with age after 60 years, except for males in High-middle and Middle SDI regions, where the age-standardized mortality rates decreased after 95 years of age. The age-standardized DALYs for PAH in all five SDI regions increased with age after 45 years, but the age-standardized DALYs for males in High-middle and Middle SDI regions declined after 95 years of age (Figure 5).

### 3.5 Regional trends

Among the 21 regions in 2021, Western Europe had the highest age-standardized prevalence rate for PAH (3.56 [2.92, 4.35] per 100,000) (Supplementary Table S1), with no differences observed after stratification by gender (Supplementary Table S2). North Africa and Middle East had the highest age-standardized mortality rate for PAH (0.44 [0.31, 0.53] per 100,000) (Supplementary Table S3). Among males, East Asia had the highest age-standardized mortality rate (0.48 [0.30, 0.60] per 100,000), while among females, North Africa and the Middle East had the highest age-standardized mortality rate (0.52 [0.34, 0.68] per 100,000) (Supplementary Table S2). North Africa and Middle East also had the highest age-standardized DALYs for PAH (14.81 [10.76, 17.96] per 100,000) (Supplementary Table S4), with no differences observed after stratification by gender (Supplementary Table S2).

Between 1990 and 2021, Western Sub-Saharan Africa experienced the largest increase in age-standardized prevalence rate for PAH (AAPC 0.66% [0.54, 0.78%]), while Central Sub-Saharan Africa had the largest decrease (AAPC −1.32% [−1.40, −1.24%]) (Supplementary Table S1). Except for Central Asia (AAPC 0.11% [−0.18, 0.40%]), all other regions showed a decline in age-standardized mortality rates, with the most significant decrease observed in Eastern Europe (AAPC −2.98% [−3.74, −2.21%]) (Supplementary Table S3). Age-standardized DALYs for PAH decreased in all regions, with the largest decline in Eastern Europe (AAPC −3.22% [−4.10, −2.34%]) (Supplementary Table S4).

### 3.6 National trends

**Figure 6:**
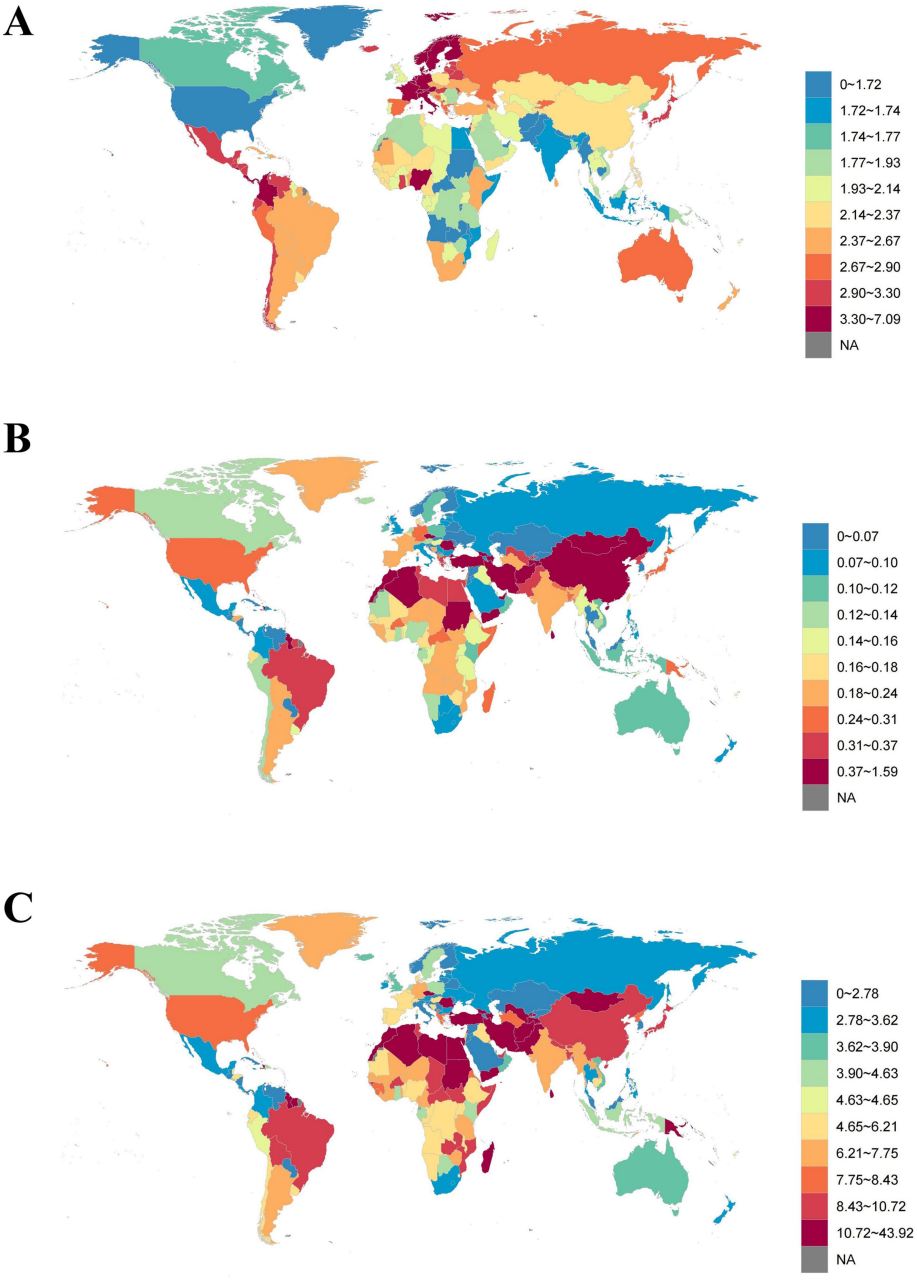
Global map of age-standardized **(A)** prevalence, **(B)** mortality, and **(C)** DALYs of PAH in 2021, per 100,000.

At the national level, Switzerland had the highest age-standardized prevalence rate for PAH in 2021 (7.09 [5.80, 8.66] per 100,000), while Mongolia had the highest age-standardized mortality rate (1.59 [0.91, 2.05] per 100,000) and age-standardized DALYs (43.92 [25.60, 56.54] per 100,000) (Figure 6 and Supplementary Tables S5–S7).

**Figure 7:**
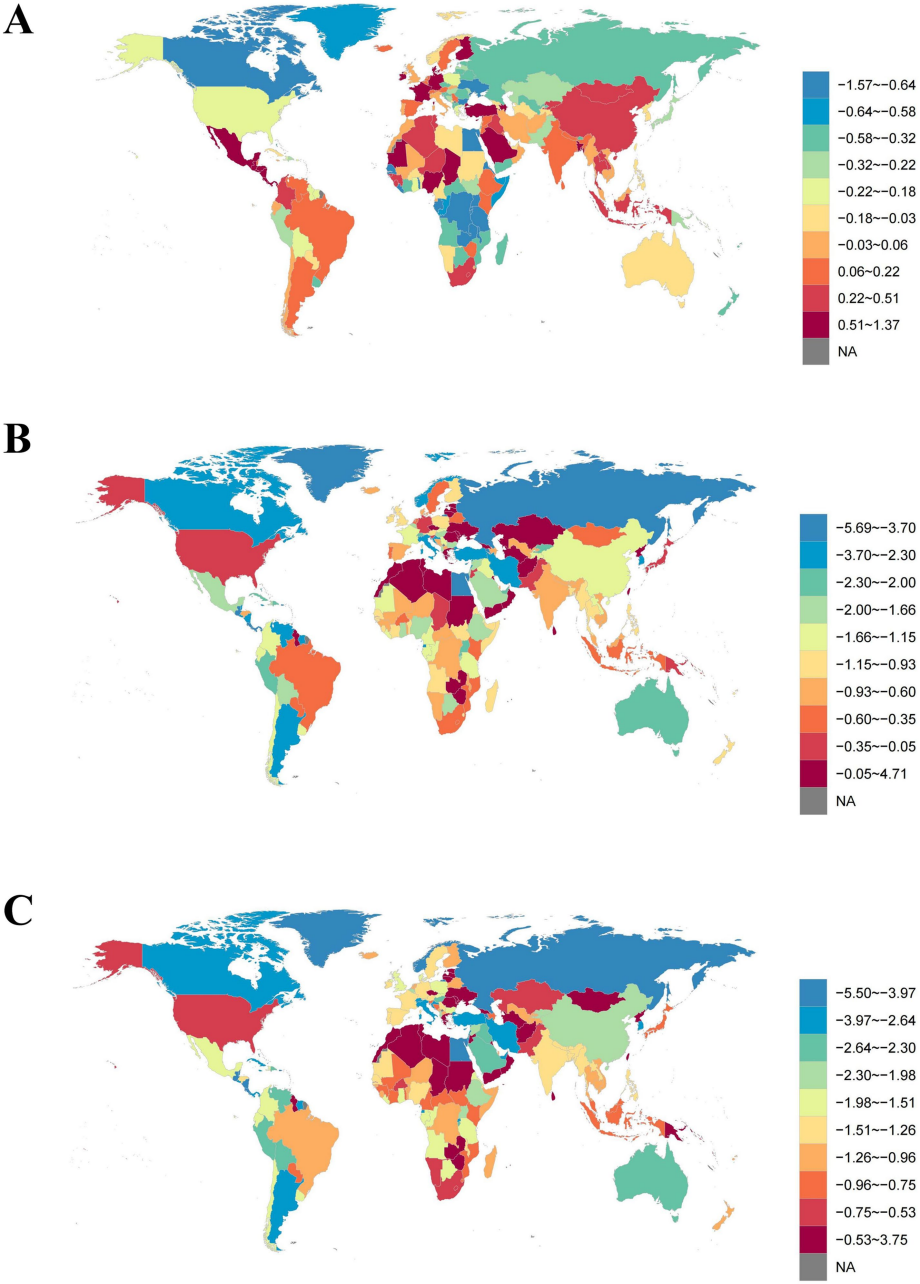
Global map of average annual percentage change in age-standardized **(A)** prevalence, **(B)** mortality, and **(C)** DALYs of PAH from 1990 to 2021.

From 1990 to 2021, the country with the largest decrease in age-standardized prevalence rate was the Democratic Republic of the Congo (AAPC −1.57% [−1.68, −1.46%]), while the country with the largest increase was Nigeria (AAPC 1.37% [1.28, 1.46%]). The country with the largest decrease in age-standardized mortality rate was Puerto Rico (AAPC −5.69% [−6.36, −5.02%]), and the country with the largest increase was Latvia (AAPC 4.71% [3.42, 6.01%]). The country with the largest decrease in age-standardized DALYs was Puerto Rico (AAPC −5.50% [−6.31, −4.69%]), and the country with the largest increase was Mauritius (AAPC 3.75% [1.92, 5.61%]) (Figure 7 and Supplementary Tables S5–S7).

### 3.7 Predictive analysis to 2022–2050

**Figure 8:**
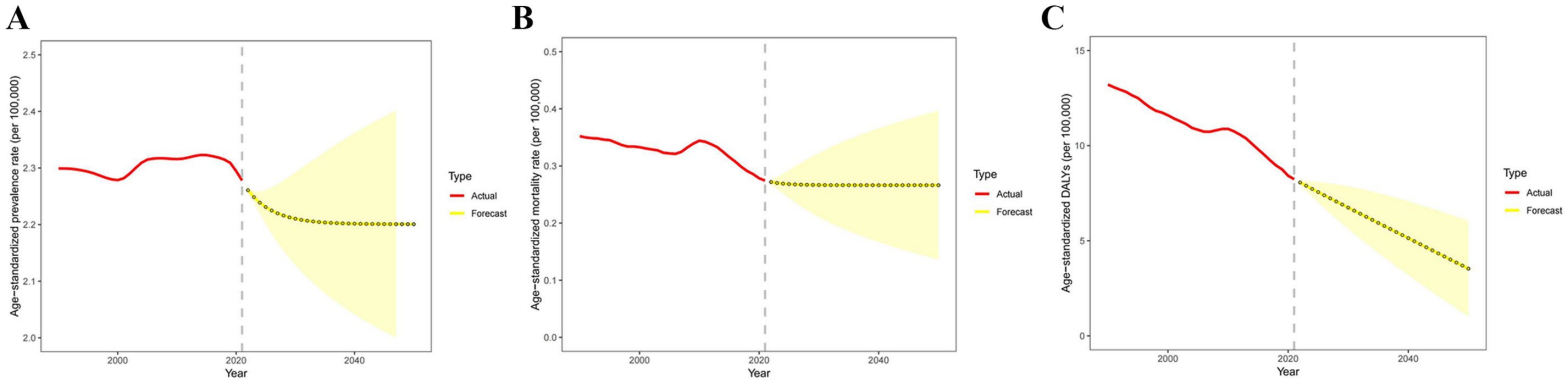
Temporal trend of predicted global age-standardized **(A)** prevalence, **(B)** mortality, and **(C)** DALYs of PAH from 2022 to 2050. DALYs, disability-adjusted life years.

By 2050, the projected global age-standardized prevalence rate for PAH is expected to be 2.20 per 100,000; the projected age-standardized mortality rate is expected to be 0.27 per 100,000; and the projected age-standardized DALYs are expected to be 3.53 per 100,000 (Figure 8 and Supplementary Table S8).

## 4 Discussion

In recent years, PAH has received widespread attention in both scientific research and clinical fields, yet it has not garnered sufficient emphasis in public health and epidemiological studies. This study conducted a comprehensive assessment of the prevalence, mortality, and DALYs associated with PAH from 1990 to 2021 at the global, regional, and national levels. Our findings reveal that, although the age-standardized prevalence rate of PAH remained relatively stable over the past 32 years, the number of patients increased significantly, while age-standardized mortality and DALYs decreased. Substantial variations exist in the epidemiological indices of PAH across different regions, socio-demographic backgrounds, age groups, and genders. To allocate health resources effectively, formulate policies, and provide targeted guidelines, it is crucial to have a comprehensive understanding of the global, regional, and national burden of PAH.

From 1990 to 2021, although the age-standardized prevalence rate fluctuated between 2.28 and 2.32 per 100,000, the number of cases increased by 81.5%. Additionally, the age-standardized mortality rate decreased by 22.9%, and DALYs declined by 37.6%. These findings are in line with previous research results (6–8, 16–18). The increase in the total number of patients with PAH can be attributed to the growth of the world’s population and the aging of the population. With the development and gradual dissemination of specific medications for PAH, the 5-year survival rate of PAH has improved globally (7, 8). However, PAH remains a severe disease with poor prognosis.

Over the past 32 years, the prevalence rate of PAH increased most significantly in Western Sub-Saharan Africa and Central Latin America. This may be attributed to the enhanced detection rates of the disease as these regions experienced economic and medical advancements. The DALYs attributed to PAH have witnessed a decline across all regions, with Eastern Europe, Southern Latin America, North Africa, and the Middle East experiencing the most pronounced reductions. Conversely, Oceania and High-income North America have seen more modest decreases. As medical infrastructure rapidly evolves in countries with less advanced economic and medical systems, the disparity in the burden posed by PAH between these nations and developed countries is progressively narrowing. In 2021, the regions with the highest prevalence of PAH were primarily Western Europe, Central Latin America, and High-income Asia Pacific. Conversely, the regions with the lowest prevalence included South Asia, High-income North America, and Oceania. Previous studies suggest that the etiology of PAH varies across different regions (19, 20), which may affect the prevalence of PAH. The regions with the highest mortality rates and DALYs globally are primarily Central Asia, North Africa and Middle East, while the regions with the lowest mortality rates and DALYs are mainly Central Latin America, Eastern Europe, and Australasia. High mortality rates for PAH are often observed in countries with lower SDI. The regional variations in prevalence, mortality, and DALYs may be attributed to differences in underlying determinants, including scarcity of medical resources, inadequate case detection, and ethnic disparities (20–25). In order to better guide policy formulation, it is necessary to understand the epidemiological characteristics of PAH in different regions and countries. At the national level, Switzerland and Sweden exhibited the highest prevalence rates of PAH, while Pakistan and Afghanistan had lower prevalence rates, which largely reflects the detection rates of PAH under different medical environments. Countries with underdeveloped medical systems urgently need to improve the detection rate of PAH and provide scientific treatment as early as possible in order to reduce the burden of PAH.

As the SDI increases, the age-standardized prevalence rate tends to rise, while the age-standardized mortality rate and age-standardized DALYs show a declining trend. In countries with low SDI, patients with PAH do not receive timely treatment, and often suffer from low medical awareness, low medical trust, low consultation rates, poor treatment outcomes, low control rates, as well as silent progression and deterioration of their condition (26, 27). People in regions with limited access to high-quality healthcare services may be less inclined to seek medical care compared to those in high-income areas.

The age-standardized prevalence rate of female patients with PAH of all ages is higher than that of males, which is similar to the results of previous studies (19, 20), indicating that being female is a risk factor for PAH (28). Sex hormones and their receptors, metabolites, immune-mediated mechanisms, genetic factors, comorbidities, and sociocultural factors may all play complex roles in the manifestation of PAH (29). Estrogen metabolites, as well as receptors and enzymes related to estrogen signaling pathways and associated conditions (such as BMPR2 mutations), contribute more specifically to the penetrance of PAH in females (28). Previous studies have shown that female patients with PAH tend to have better right ventricular function and consequently better prognosis compared to males (30, 31). However, this study indicates that while the 1990 data suggested lower mortality and DALYs from PAH in females compared to males, the annual average decline in mortality and DALYs was more significant in males. The 2021 data on the burden of PAH did not suggest that female patients with PAH have better prognosis than males. The physiological, pathological, and epidemiological differences between males and females suggest the need to consider further gender-specific diagnostic and treatment strategies in order to improve patient outcomes.

The 2021 data shows that the age-standardized prevalence rate of PAH increases with age, peaking at around 80 years old and then declining. The age-standardized mortality rate and DALYs for PAH is lowest between 5 and 14 years of age, and thereafter, both the age-standardized mortality rate and DALYs increase with age, which is consistent with previous studies (18, 25). The 5-year survival rate for elderly patients with PAH is significantly lower than that of patients aged 18–45 years (32). The high mortality risk among elderly patients with PAH may be related to multiple factors, including their basic physical condition, comorbidities, unique disease phenotypes, different treatment modalities, and diminished response to targeted therapies for PAH (33). Over the past three decades, the age-standardized mortality rate and DALYs of PAH in individuals aged 0–84 years have shown a declining trend, but an increasing trend has been observed in those over 85 years old. The high prevalence of PAH among the elderly and its severe detrimental impact on prognosis should be given significant attention. There is an urgent need to implement effective strategies to identify high-risk elderly patients with PAH, modify risk factors, and facilitate targeted early diagnosis and treatment of this condition.

Our projections indicate that the age-standardized prevalence rate of PAH will gradually decline after 2021. By 2050, the expected age-standardized prevalence rate for global PAH is projected to be 2.20 per 100,000. However, mortality rates are not anticipated to change significantly, with an expected age-standardized mortality rate of 0.27 per 100,000 in 2050. In contrast, DALYs are projected to decrease rapidly after 2021, with an estimated age-standardized DALYs of 3.53 per 100,000 in 2050.

The strength of this study lies in its comprehensive epidemiological analysis of global PAH trends based on the 2021 GBD results, filling a gap in this field. This study not only encompasses the three classic indicators of prevalence, mortality, and DALYs and their trends at the global, regional, and national levels, but it also reports on the influence of age, gender, year, and sociodemographic index on the burden of PAH. Furthermore, this study forecasts the global burden trends of PAH from 2022 to 2050, offering valuable insights into the future trajectory of this disease.

This study has several limitations. Firstly, our analysis relies on the latest data from the GBD 2021 study, which integrates estimates of PAH prevalence, mortality, DALYs, and other metrics from various input sources. However, due to differences in methodology and data sources, there may be discrepancies between the GBD estimates and other national and local data. Secondly, there is a time lag in the availability of disease burden data, and it is essential for us to focus on the latest global PAH data promptly thereafter. Thirdly, potential biases may still exist in the data sources. Differences in healthcare systems and related policies across countries may influence the results of disease burden estimates as well as predictions for future disease burdens.

## 5 Conclusion

In summary, from 1990 to 2021, although the age-standardized prevalence rate of PAH globally remained relatively stable, the number of cases continued to rise. Women and the elderly are high-risk populations for PAH, with elderly PAH patients having the worst prognosis. The overall trend of PAH burden shows regional and national differences. Therefore, it is necessary to implement targeted interventions and public health initiatives to alleviate the burden of PAH.

## Data Availability

The original contributions presented in the study are included in the article/Supplementary material, further inquiries can be directed to the corresponding authors.

## Footnotes

^1^ https://vizhub.healthdata.org/gbd-results
