# Supplementary material for: Trends and levels of the global, regional, and national burden of pulmonary arterial hypertension from 1990 to 2021: findings from the global burden of disease study 2021
Source: Front Med (Lausanne). 2024 Dec 10;11:1515961. doi: 10.3389/fmed.2024.1515961 (PMC11666447; doi:10.3389/fmed.2024.1515961)
Supplement: Supplementary file 1 [file Data_Sheet_1.DOCX]

Supplementary Material

# Supplementary Tables

**Supplementary Table S1** Age-standardized prevalence rate and AAPC of PAH at regional level, 1990-2021.

| Regions | Prevalence (95% UI) | | | | |
| --- | --- | --- | --- | --- | --- |
|  | Cases in 1990 | Age-standardized rate in 1990 (per 100,000) | Cases in 2021 | Age-standardized rate in 2021 (per 100,000) | 1990–2021 AAPC (95% CI) |
| East Asia | 22867 (18513,28351) | 2.07 (1.68,2.55) | 42486(33930,53043) | 2.23 (1.81,2.75) | 0.24 (0.22, 0.26) |
| Southeast Asia | 6366 (5234,7852) | 1.77 (1.43,2.17) | 13616(11019,16957) | 1.90 (1.54,2.32) | 0.23 (0.18, 0.27) |
| Oceania | 92 (75,112) | 1.98 (1.60,2.40) | 196 (159,241) | 1.80 (1.45,2.17) | -0.29 (-0.35, -0.23) |
| Central Asia | 1409 (1145,1727) | 2.46 (2.01,3.00) | 2162 (1758,2677) | 2.33 (1.90,2.85) | -0.17 (-0.22, -0.13) |
| Central Europe | 3456 (2821,4228) | 2.52 (2.08,3.05) | 3521 (2864,4311) | 2.30 (1.89,2.79) | -0.28 (-0.32, -0.23) |
| Eastern Europe | 8179 (6670,10049) | 3.21 (2.65,3.91) | 7686 (6264,9489) | 2.83 (2.32,3.43) | -0.40 (-0.48, -0.31) |
| High-income Asia Pacific | 6369 (5212,7812) | 3.25 (2.67,3.97) | 9167 (7427,11362) | 3.01 (2.47,3.68) | -0.24 (-0.27, -0.21) |
| Australasia | 670 (544,828) | 2.99 (2.44,3.70) | 1167 (936,1444) | 2.83 (2.29,3.44) | -0.18 (-0.19, -0.17) |
| Western Europe | 15675 (12582,19294) | 3.22 (2.62,3.95) | 23621(19121,29197) | 3.56 (2.92,4.35) | 0.33 (0.27, 0.39) |
| Southern Latin America | 1281 (1040,1574) | 2.69 (2.18,3.30) | 2227 (1800,2760) | 2.82 (2.29,3.48) | 0.15 (0.12, 0.18) |
| High-income North America | 5984 (4856,7325) | 1.87 (1.53,2.29) | 8625 (6918,10745) | 1.73 (1.40,2.12) | -0.26 (-0.29, -0.23) |
| Caribbean | 778 (635,954) | 2.56 (2.08,3.13) | 1229 (987,1506) | 2.39 (1.93,2.93) | -0.23 (-0.26, -0.19) |
| Andean Latin America | 812 (667,997) | 2.91 (2.35,3.58) | 1777 (1434,2194) | 2.77 (2.24,3.40) | -0.16 (-0.19, -0.14) |
| Central Latin America | 3262 (2679,3991) | 2.71 (2.21,3.30) | 8421 (6856,10383) | 3.25 (2.64,3.99) | 0.58 (0.52, 0.64) |
| Tropical Latin America | 2885 (2357,3541) | 2.35 (1.90,2.88) | 6239 (5020,7679) | 2.48 (2.00,3.04) | 0.18 (0.09, 0.26) |
| North Africa and Middle East | 4968 (4056,6153) | 2.02 (1.64,2.49) | 11591 (9389,14436) | 2.03 (1.64,2.49) | -0.01 (-0.08, 0.06) |
| South Asia | 12897 (10582,15994) | 1.58 (1.28,1.93) | 29561 (23835,36927) | 1.71 (1.38,2.09) | 0.25 (0.23, 0.27) |
| Central Sub-Saharan Africa | 972 (795,1206) | 2.79 (2.26,3.44) | 1693 (1378,2094) | 1.86 (1.49,2.24) | -1.32 (-1.40, -1.24) |
| Eastern Sub-Saharan Africa | 2875 (2359,3536) | 2.40 (1.94,2.92) | 5907 (4822,7344) | 2.09 (1.70,2.53) | -0.46 (-0.50, -0.41) |
| Southern Sub-Saharan Africa | 796 (658,967) | 2.12 (1.73,2.56) | 1596 (1287,1945) | 2.28 (1.85,2.76) | 0.21 (0.18, 0.24) |
| Western Sub-Saharan Africa | 3112 (2565,3829) | 2.42 (1.96,2.97) | 9319 (7635,11570) | 2.97 (2.40,3.63) | 0.66 (0.54, 0.78) |

Note: AAPC, average annual percentage change; CI, confidence interval; UI, uncertainty interval.

**Supplementary Table S2** Age-standardized prevalence, mortality and DALYs of PAH in 2021 at regional levels by sex.

| Regions | Age-standardized rate in 2021 (per 100,000) | | | | | |
| --- | --- | --- | --- | --- | --- | --- |
|  | Male | | | Female | | |
|  | Prevalence (95% UI) | Mortality (95% UI) | DALYs (95% UI) | Prevalence (95% UI) | Mortality (95% UI) | DALYs (95% UI) |
| East Asia | 1.90 (1.53,2.33) | 0.48 (0.30,0.60) | 9.29 (5.95,11.75) | 2.57 (2.08,3.18) | 0.37 (0.23,0.49) | 8.71 (5.68,12.56) |
| Southeast Asia | 1.64 (1.33,1.99) | 0.09 (0.06,0.16) | 3.67 (2.79,7.02) | 2.14 (1.73,2.63) | 0.14 (0.09,0.49) | 5.58 (3.94,13.19) |
| Oceania | 1.44 (1.16,1.76) | 0.21 (0.13,0.48) | 8.05 (5.56,15.92) | 2.18 (1.76,2.63) | 0.28 (0.16,0.61) | 12.35 (6.92,22.58) |
| Central Asia | 1.79 (1.45,2.19) | 0.47 (0.39,0.56) | 14.15(11.7,17.37) | 2.82 (2.31,3.44) | 0.38 (0.28,0.48) | 11.92 (8.20,15.30) |
| Central Europe | 1.90 (1.55,2.29) | 0.19 (0.18,0.21) | 5.16 (4.70,5.68) | 2.68 (2.21,3.26) | 0.23 (0.20,0.26) | 6.97 (6.21,7.80) |
| Eastern Europe | 2.20 (1.80,2.67) | 0.12 (0.11,0.13) | 3.80 (3.47,4.14) | 3.34 (2.73,4.05) | 0.08 (0.07,0.08) | 3.04 (2.72,3.38) |
| High-income Asia Pacific | 2.35 (1.92,2.89) | 0.19 (0.17,0.21) | 6.86 (6.36,7.49) | 3.66 (3.00,4.52) | 0.26 (0.23,0.30) | 9.72 (8.88,10.57) |
| Australasia | 2.17 (1.74,2.63) | 0.08 (0.07,0.09) | 2.44 (2.20,2.74) | 3.47 (2.83,4.23) | 0.15 (0.12,0.16) | 4.85 (4.41,5.30) |
| Western Europe | 2.64 (2.15,3.21) | 0.14 (0.13,0.15) | 3.97 (3.75,4.20) | 4.46 (3.65,5.49) | 0.21 (0.18,0.23) | 6.06 (5.62,6.44) |
| Southern Latin America | 2.14 (1.72,2.59) | 0.13 (0.12,0.14) | 4.55 (4.16,5.00) | 3.44 (2.80,4.25) | 0.23 (0.21,0.25) | 8.41 (7.82,9.09) |
| High-income North America | 1.26 (1.02,1.54) | 0.21 (0.20,0.23) | 5.93 (5.56,6.27) | 2.17 (1.75,2.67) | 0.35 (0.30,0.38) | 9.29 (8.57,9.89) |
| Caribbean | 1.79 (1.44,2.18) | 0.20 (0.12,0.28) | 12.69 (6.12,20.00) | 2.95 (2.39,3.64) | 0.20 (0.13,0.33) | 10.64 (6.28,20.25) |
| Andean Latin America | 2.10 (1.69,2.57) | 0.13 (0.10,0.16) | 4.73 (3.66,6.01) | 3.42 (2.76,4.19) | 0.18 (0.14,0.24) | 6.70 (5.09,8.91) |
| Central Latin America | 2.41 (1.96,2.94) | 0.06 (0.06,0.07) | 2.34 (2.03,2.76) | 4.00 (3.26,4.92) | 0.10 (0.09,0.12) | 3.60 (3.13,4.20) |
| Tropical Latin America | 1.87 (1.52,2.26) | 0.25 (0.23,0.27) | 8.00 (7.43,8.57) | 3.03 (2.44,3.70) | 0.37 (0.34,0.40) | 12.23(11.44,12.93) |
| North Africa and Middle East | 1.53 (1.24,1.88) | 0.36 (0.24,0.43) | 14.35 (9.50,17.34) | 2.56 (2.08,3.14) | 0.52 (0.34,0.68) | 15.24 (10.46,20.11) |
| South Asia | 1.29 (1.04,1.58) | 0.24 (0.14,0.48) | 7.87 (5.49,12.88) | 2.13 (1.72,2.62) | 0.27 (0.14,0.44) | 9.22 (4.94,13.10) |
| Central Sub-Saharan Africa | 1.42 (1.13,1.72) | 0.22 (0.09,0.43) | 7.02 (3.11,11.92) | 2.25 (1.81,2.73) | 0.17 (0.07,0.45) | 5.40 (2.64,13.08) |
| Eastern Sub-Saharan Africa | 1.59 (1.29,1.93) | 0.22 (0.08,0.43) | 7.99 (3.59,14.66) | 2.55 (2.07,3.10) | 0.15 (0.06,0.43) | 5.76 (2.84,15.85) |
| Southern Sub-Saharan Africa | 1.74 (1.41,2.10) | 0.14 (0.11,0.17) | 5.14 (3.98,6.54) | 2.72 (2.21,3.30) | 0.09 (0.06,0.12) | 3.66 (2.40,4.61) |
| Western Sub-Saharan Africa | 2.15 (1.74,2.64) | 0.20 (0.08,0.38) | 8.13 (4.45,12.33) | 3.71 (3.01,4.54) | 0.13 (0.06,0.30) | 4.87 (2.82,9.83) |

Note: DALYs, disability-adjusted life years; UI, uncertainty interval.

**Supplementary Table S3** Age-standardized mortality rate and AAPC of PAH at regional level, 1990-2021.

| Regions | Mortality (95% UI) | | | | |
| --- | --- | --- | --- | --- | --- |
|  | Cases in 1990 | Age-standardized rate in 1990 (per 100,000) | Cases in 2021 | Age-standardized rate in 2021 (per 100,000) | 1990–2021 AAPC (95% CI) |
| East Asia | 4115 (3141,5526) | 0.59 (0.45,0.81) | 7490 (4986,9266) | 0.41 (0.28,0.50) | -1.23 (-1.49, -0.96) |
| Southeast Asia | 506 (340,1094) | 0.15 (0.09,0.43) | 741 (525,1850) | 0.12 (0.08,0.32) | -0.76 (-0.79, -0.72) |
| Oceania | 12 (8,19) | 0.28 (0.18,0.53) | 25 (17,43) | 0.24 (0.16,0.48) | -0.47 (-0.55, -0.38) |
| Central Asia | 208 (163,240) | 0.40 (0.31,0.47) | 319 (261,382) | 0.41 (0.34,0.48) | 0.11 (-0.18, 0.40) |
| Central Europe | 355 (309,394) | 0.26 (0.22,0.28) | 438 (398,479) | 0.21 (0.19,0.23) | -0.70 (-0.96, -0.43) |
| Eastern Europe | 563 (512,651) | 0.24 (0.22,0.27) | 278 (258,300) | 0.09 (0.08,0.10) | -2.98 (-3.74, -2.21) |
| High-income Asia Pacific | 434 (410,459) | 0.26 (0.24,0.27) | 1049 (826,1201) | 0.23 (0.20,0.26) | -0.34 (-0.61, -0.06) |
| Australasia | 45 (39,57) | 0.21 (0.18,0.26) | 58 (49,65) | 0.11 (0.10,0.13) | -1.85 (-2.22, -1.49) |
| Western Europe | 1233 (1094,1380) | 0.24 (0.21,0.27) | 1788 (1533,1943) | 0.18 (0.16,0.19) | -0.94 (-1.19, -0.68) |
| Southern Latin America | 169 (151,186) | 0.36 (0.32,0.40) | 150 (138,162) | 0.18 (0.17,0.20) | -2.17 (-2.47, -1.87) |
| High-income North America | 1064 (947,1167) | 0.32 (0.28,0.35) | 1880 (1620,2043) | 0.29 (0.26,0.31) | -0.26 (-0.43, -0.09) |
| Caribbean | 124 (83,169) | 0.38 (0.28,0.49) | 95 (66,130) | 0.20 (0.13,0.29) | -1.96 (-2.19, -1.73) |
| Andean Latin America | 84 (56,112) | 0.28 (0.21,0.35) | 91 (72,119) | 0.16 (0.12,0.20) | -1.83 (-2.14, -1.51) |
| Central Latin America | 180 (157,210) | 0.16 (0.14,0.19) | 201 (177,230) | 0.08 (0.07,0.10) | -2.13 (-2.36, -1.90) |
| Tropical Latin America | 394 (373,412) | 0.37 (0.35,0.39) | 779 (714,822) | 0.32 (0.29,0.34) | -0.50 (-0.78, -0.23) |
| North Africa and Middle East | 2142 (1309,2739) | 0.77 (0.56,1.00) | 1896 (1328,2305) | 0.44 (0.31,0.53) | -1.75 (-1.90, -1.61) |
| South Asia | 2385 (1502,3418) | 0.31 (0.17,0.50) | 3549 (2321,5532) | 0.25 (0.16,0.42) | -0.63 (-0.88, -0.39) |
| Central Sub-Saharan Africa | 87 (55,163) | 0.24 (0.11,0.47) | 131 (62,237) | 0.19 (0.08,0.37) | -0.76 (-0.80, -0.73) |
| Eastern Sub-Saharan Africa | 366 (217,687) | 0.27 (0.12,0.52) | 468 (219,878) | 0.18 (0.07,0.34) | -1.37 (-1.44, -1.29) |
| Southern Sub-Saharan Africa | 43 (32,58) | 0.12 (0.08,0.17) | 72 (53,86) | 0.11 (0.08,0.13) | -0.26 (-0.40, -0.12) |
| Western Sub-Saharan Africa | 335 (195,616) | 0.25 (0.09,0.51) | 523 (306,774) | 0.17 (0.07,0.28) | -1.29 (-1.35, -1.23) |

Note: AAPC, average annual percentage change; CI, confidence interval; UI, uncertainty interval.

**Supplementary Table S4** Age-standardized DALYs and AAPC of PAH at regional level, 1990-2021.

| Regions | DALYs (95% UI) | | | | |
| --- | --- | --- | --- | --- | --- |
|  | Cases in 1990 | Age-standardized rate in 1990 (per 100,000) | Cases in 2021 | Age-standardized rate in 2021 (per 100,000) | 1990–2021 AAPC (95% CI) |
| East Asia | 151596 (117394,205773) | 15.78 (12.34,21.21) | 154740 (102939,190399) | 8.84 (5.99,11.01) | -1.94 (-2.38, -1.49) |
| Southeast Asia | 27786 (18242,51943) | 6.31 (4.28,12.72) | 31112 (22913,58708) | 4.65 (3.39,9.25) | -0.97 (-1.01, -0.93) |
| Oceania | 704 (448,1090) | 10.85 (7.02,17.55) | 1455 (988,2411) | 10.14 (6.90,17.10) | -0.20 (-0.42, 0.02) |
| Central Asia | 9071 (7195,10646) | 14.14 (11.27,16.45) | 11619 (9514,14202) | 12.91 (10.61,15.60) | -0.36 (-0.64, -0.07) |
| Central Europe | 11026 (9784,12154) | 8.15 (7.25,8.95) | 10424 (9512,11459) | 6.05 (5.50,6.67) | -1.02 (-1.31, -0.73) |
| Eastern Europe | 20628 (18781,23860) | 9.23 (8.47,10.49) | 8357 (7760,8996) | 3.32 (3.10,3.56) | -3.22 (-4.10, -2.34) |
| High-income Asia Pacific | 18474 (17689,19467) | 12.03 (11.43,12.79) | 19988 (17442,21997) | 8.31 (7.74,8.87) | -1.19 (-1.65, -0.73) |
| Australasia | 1514 (1337,1856) | 7.37 (6.49,9.06) | 1434 (1305,1563) | 3.67 (3.38,3.99) | -2.18 (-2.53, -1.84) |
| Western Europe | 34599 (31743,38281) | 8.27 (7.69,9.18) | 34043 (31024,36440) | 5.05 (4.75,5.32) | -1.56 (-1.77, -1.35) |
| Southern Latin America | 7983 (7181,8803) | 16.28 (14.65,17.95) | 4739 (4439,5092) | 6.55 (6.13,7.07) | -3.10 (-3.36, -2.85) |
| High-income North America | 30206 (27736,32715) | 10.13 (9.39,10.99) | 38373 (35060,40844) | 7.71 (7.17,8.18) | -0.87 (-1.11, -0.62) |
| Caribbean | 7398 (4170,11145) | 19.72 (11.78,28.82) | 5071 (2988,7877) | 11.73 (6.48,18.74) | -1.60 (-1.82, -1.38) |
| Andean Latin America | 5049 (2977,7422) | 11.92 (7.83,16.43) | 3544 (2795,4490) | 5.73 (4.52,7.28) | -2.24 (-2.44, -2.04) |
| Central Latin America | 9966 (8844,11855) | 6.12 (5.40,7.16) | 7246 (6391,8407) | 3.00 (2.63,3.51) | -2.26 (-2.54, -1.97) |
| Tropical Latin America | 19065 (17856,20357) | 14.18 (13.35,14.98) | 24235 (23002,25403) | 10.22 (9.65,10.78) | -1.06 (-1.42, -0.70) |
| North Africa and Middle East | 145728 (74879,204070) | 35.84 (21.25,46.14) | 80753 (58086,98810) | 14.81 (10.76,17.96) | -2.78 (-2.93, -2.63) |
| South Asia | 136086 (77808,184381) | 12.24 (7.79,17.12) | 136563 (97809,189353) | 8.54 (6.02,12.46) | -1.12 (-1.32, -0.93) |
| Central Sub-Saharan Africa | 5367 (3258,10525) | 9.09 (5.65,17.13) | 6524 (3586,11025) | 6.16 (2.94,11.09) | -1.25 (-1.30, -1.21) |
| Eastern Sub-Saharan Africa | 23148 (14694,45765) | 11.11 (6.18,21.17) | 26605 (14265,49615) | 6.85 (3.30,12.62) | -1.54 (-1.61, -1.47) |
| Southern Sub-Saharan Africa | 2188 (1667,2875) | 4.70 (3.65,6.37) | 3212 (2359,3891) | 4.33 (3.21,5.20) | -0.29 (-0.48, -0.09) |
| Western Sub-Saharan Africa | 19834 (13553,38853) | 9.34 (5.22,16.94) | 32071 (22084,47222) | 6.50 (3.77,9.47) | -1.16 (-1.21, -1.11) |

Note: AAPC, average annual percentage change; CI, confidence interval; UI, uncertainty interval; DALYs, disability-adjusted life years

**Supplementary T****able S5** Age-standardized prevalence rate and AAPC of PAH in 204 countries and territories, 1990-2021.

|  | Prevalence (95% UI) | | | | |
| --- | --- | --- | --- | --- | --- |
|  | Cases in 1990 | Age-standardized rate in 1990 (per 100,000) | Cases in 2021 | Age-standardized rate in 2021 (per 100,000) | 1990–2021 AAPC (95% CI) |
| China | 22028 (17840,27321) | 2.07 (1.68,2.54) | 41135 (32839,51357) | 2.24 (1.81,2.75) | 0.25 (0.23, 0.28) |
| Democratic People's Republic of Korea | 386 (311,478) | 2.03 (1.63,2.48) | 602 (479,752) | 1.93 (1.56,2.37) | -0.16 (-0.18, -0.14) |
| Taiwan (Province of China) | 454 (368,559) | 2.37 (1.93,2.88) | 749 (599,929) | 2.26 (1.83,2.77) | -0.15 (-0.16, -0.14) |
| Cambodia | 110 (90,137) | 1.60 (1.29,1.95) | 245 (197,301) | 1.60 (1.29,1.94) | -0.01 (-0.03, 0.02) |
| Indonesia | 2131 (1732,2637) | 1.49 (1.20,1.82) | 4923 (3971,6154) | 1.73 (1.41,2.12) | 0.51 (0.45, 0.56) |
| Lao People's Democratic Republic | 51 (41,63) | 1.73 (1.40,2.13) | 126 (103,157) | 2.02 (1.64,2.44) | 0.50 (0.46, 0.55) |
| Malaysia | 257 (211,322) | 1.86 (1.52,2.28) | 594 (479,737) | 1.85 (1.5,2.260) | -0.02 (-0.04, -0.00) |
| Maldives | 3 (2,3) | 1.74 (1.40,2.15) | 11 (8,14) | 2.06 (1.67,2.55) | 0.55 (0.47, 0.64) |
| Myanmar | 485 (395,595) | 1.56 (1.26,1.91) | 858 (681,1059) | 1.58 (1.27,1.93) | 0.04 (0.02, 0.07) |
| Philippines | 1047 (859,1291) | 2.31 (1.87,2.82) | 2238 (1836,2776) | 2.20 (1.79,2.68) | -0.16 (-0.20, -0.12) |
| Sri Lanka | 347 (285,427) | 2.40 (1.94,2.92) | 646 (514,798) | 2.54 (2.05,3.09) | 0.19 (0.17, 0.21) |
| Thailand | 922 (747,1145) | 1.93 (1.56,2.35) | 1892 (1513,2392) | 2.08 (1.68,2.55) | 0.24 (0.22, 0.26) |
| Timor-Leste | 9 (7,11) | 1.73 (1.41,2.13) | 20 (16,24) | 1.81 (1.47,2.21) | 0.14 (0.12, 0.16) |
| Viet Nam | 963 (787,1191) | 1.84 (1.50,2.28) | 1990 (1591,2491) | 1.87 (1.51,2.30) | 0.04 (0.02, 0.07) |
| Fiji | 13 (11,16) | 2.23 (1.82,2.73) | 17 (14,21) | 1.94 (1.58,2.37) | -0.43 (-0.52, -0.34) |
| Kiribati | 1 (1,1) | 1.81 (1.47,2.20) | 2 (1,2) | 1.66 (1.33,2.04) | -0.25 (-0.32, -0.19) |
| Marshall Islands | 1 (0,1) | 1.76 (1.44,2.15) | 1 (1,1) | 1.62 (1.31,1.95) | -0.23 (-0.30, -0.16) |
| Micronesia (Federated States of) | 1 (1,2) | 1.84 (1.50,2.22) | 2 (1,2) | 1.68 (1.37,2.03) | -0.27 (-0.33, -0.21) |
| Papua New Guinea | 55 (45,67) | 1.92 (1.56,2.36) | 141 (113,174) | 1.78 (1.43,2.16) | -0.22 (-0.29, -0.16) |
| Samoa | 3 (2,3) | 2.38 (1.93,2.92) | 4 (3,5) | 2.11 (1.71,2.56) | -0.39 (-0.44, -0.33) |
| Solomon Islands | 4 (3,5) | 1.80 (1.46,2.20) | 9 (7,11) | 1.68 (1.35,2.03) | -0.20 (-0.26, -0.14) |
| Tonga | 2 (1,2) | 2.28 (1.85,2.81) | 2 (2,2) | 2.13 (1.72,2.61) | -0.20 (-0.27, -0.13) |
| Vanuatu | 2 (1,2) | 1.65 (1.33,2.03) | 4 (3,5) | 1.56 (1.27,1.88) | -0.17 (-0.23, -0.10) |
| Armenia | 90 (74,110) | 2.87 (2.36,3.49) | 101 (81,125) | 2.77 (2.27,3.40) | -0.11 (-0.13, -0.10) |
| Azerbaijan | 138 (113,172) | 2.21 (1.81,2.69) | 299 (242,370) | 2.67 (2.19,3.25) | 0.61 (0.53, 0.69) |
| Georgia | 157 (127,192) | 2.66 (2.17,3.23) | 118 (96,145) | 2.59 (2.12,3.14) | -0.09 (-0.10, -0.08) |
| Kazakhstan | 368 (298,445) | 2.51 (2.04,3.04) | 442 (361,545) | 2.33 (1.90,2.83) | -0.25 (-0.34, -0.17) |
| Kyrgyzstan | 88 (73,106) | 2.42 (2.00,2.95) | 164 (134,201) | 2.72 (2.22,3.33) | 0.39 (0.33, 0.44) |
| Mongolia | 28 (23,35) | 1.80 (1.47,2.22) | 63 (51,78) | 2.09 (1.69,2.55) | 0.49 (0.46, 0.51) |
| Tajikistan | 90 (72,113) | 2.35 (1.91,2.88) | 192 (155,239) | 2.27 (1.84,2.77) | -0.10 (-0.12, -0.08) |
| Turkmenistan | 61 (50,76) | 2.23 (1.81,2.73) | 103 (84,128) | 2.15 (1.76,2.64) | -0.12 (-0.14, -0.10) |
| Uzbekistan | 388 (317,476) | 2.48 (2.03,3.03) | 680 (555,843) | 2.10 (1.72,2.55) | -0.51 (-0.58, -0.44) |
| Albania | 85 (68,105) | 3.09 (2.52,3.77) | 91 (73,111) | 2.68 (2.20,3.28) | -0.44 (-0.47, -0.41) |
| Bosnia and Herzegovina | 140 (114,172) | 3.09 (2.54,3.74) | 121 (98,150) | 2.80 (2.31,3.41) | -0.31 (-0.34, -0.28) |
| Bulgaria | 255 (208,312) | 2.47 (2.05,2.97) | 181 (146,222) | 1.93 (1.57,2.36) | -0.79 (-0.85, -0.73) |
| Croatia | 163 (131,200) | 2.94 (2.43,3.56) | 150 (121,183) | 2.60 (2.13,3.13) | -0.38 (-0.39, -0.36) |
| Czechia | 350 (286,425) | 2.94 (2.44,3.55) | 375 (303,459) | 2.62 (2.16,3.16) | -0.36 (-0.39, -0.32) |
| Hungary | 409 (334,501) | 3.32 (2.77,4.00) | 397 (319,484) | 3.02 (2.48,3.65) | -0.30 (-0.41, -0.19) |
| North Macedonia | 52 (42,64) | 2.61 (2.14,3.14) | 63 (51,78) | 2.33 (1.90,2.84) | -0.36 (-0.38, -0.33) |
| Montenegro | 16 (13,19) | 2.51 (2.06,3.04) | 18 (15,23) | 2.39 (1.96,2.90) | -0.15 (-0.19, -0.12) |
| Poland | 949 (777,1161) | 2.32 (1.91,2.80) | 1091 (890,1343) | 2.19 (1.80,2.65) | -0.19 (-0.22, -0.15) |
| Romania | 562 (456,686) | 2.20 (1.81,2.66) | 481 (389,592) | 1.89 (1.54,2.31) | -0.46 (-0.48, -0.45) |
| Serbia | 208 (168,258) | 1.97 (1.60,2.41) | 242 (193,300) | 2.09 (1.70,2.55) | 0.19 (0.14, 0.24) |
| Slovakia | 147 (121,180) | 2.61 (2.16,3.17) | 185 (149,232) | 2.63 (2.15,3.23) | 0.04 (-0.09, 0.17) |
| Slovenia | 65 (54,80) | 2.93 (2.42,3.53) | 74 (60,91) | 2.58 (2.12,3.14) | -0.40 (-0.44, -0.37) |
| Belarus | 433 (356,531) | 3.67 (3.04,4.46) | 385 (311,480) | 3.10 (2.53,3.78) | -0.54 (-0.73, -0.35) |
| Estonia | 66 (54,81) | 3.59 (2.97,4.36) | 57 (45,70) | 3.08 (2.53,3.73) | -0.48 (-0.61, -0.36) |
| Latvia | 110 (90,134) | 3.49 (2.87,4.24) | 87 (71,108) | 3.18 (2.62,3.86) | -0.30 (-0.36, -0.25) |
| Lithuania | 138 (113,168) | 3.33 (2.77,4.04) | 116 (94,142) | 2.94 (2.42,3.55) | -0.39 (-0.46, -0.33) |
| Republic of Moldova | 142 (117,174) | 3.17 (2.62,3.85) | 139 (112,172) | 2.93 (2.39,3.56) | -0.26 (-0.35, -0.17) |
| Russian Federation | 5368 (4377,6596) | 3.20 (2.64,3.91) | 5453 (4432,6731) | 2.90 (2.38,3.51) | -0.32 (-0.38, -0.26) |
| Ukraine | 1922 (1563,2367) | 3.11 (2.54,3.80) | 1451 (1164,1795) | 2.52 (2.06,3.07) | -0.69 (-0.74, -0.63) |
| Brunei Darussalam | 4 (3,5) | 2.08 (1.69,2.54) | 9 (7,11) | 2.02 (1.62,2.49) | -0.10 (-0.13, -0.07) |
| Japan | 5141 (4190,6322) | 3.33 (2.72,4.06) | 6754 (5460,8398) | 3.09 (2.54,3.79) | -0.23 (-0.25, -0.21) |
| Republic of Korea | 1152 (954,1431) | 3.02 (2.49,3.68) | 2234 (1798,2797) | 2.91 (2.39,3.58) | -0.11 (-0.14, -0.09) |
| Singapore | 72 (58,90) | 2.50 (2.03,3.07) | 171 (137,212) | 2.24 (1.83,2.77) | -0.35 (-0.38, -0.32) |
| Australia | 561 (458,695) | 3.01 (2.46,3.72) | 992 (794,1226) | 2.87 (2.33,3.49) | -0.15 (-0.16, -0.14) |
| New Zealand | 108 (87,133) | 2.92 (2.36,3.56) | 175 (141,218) | 2.62 (2.11,3.21) | -0.34 (-0.36, -0.32) |
| Andorra | 2 (2,3) | 3.45 (2.82,4.22) | 5 (4,6) | 3.66 (2.98,4.47) | 0.19 (0.14, 0.25) |
| Austria | 341 (273,421) | 3.42 (2.76,4.19) | 481 (387,595) | 3.57 (2.91,4.41) | 0.13 (0.08, 0.18) |
| Belgium | 512 (412,632) | 3.96 (3.24,4.88) | 720 (585,900) | 4.22 (3.48,5.21) | 0.22 (0.18, 0.26) |
| Cyprus | 30 (24,37) | 3.61 (2.92,4.42) | 76 (62,93) | 4.24 (3.49,5.13) | 0.52 (0.47, 0.57) |
| Denmark | 206 (166,255) | 3.12 (2.54,3.85) | 350 (282,438) | 4.03 (3.31,4.97) | 0.83 (0.78, 0.88) |
| Finland | 197 (158,244) | 3.16 (2.56,3.88) | 332 (268,415) | 3.79 (3.10,4.61) | 0.57 (0.48, 0.67) |
| France | 2329 (1884,2879) | 3.31 (2.67,4.09) | 4014 (3244,4928) | 4.07 (3.34,4.98) | 0.66 (0.61, 0.70) |
| Germany | 3021 (2399,3717) | 2.88 (2.34,3.51) | 4886 (3954,6094) | 3.59 (2.93,4.36) | 0.71 (0.45, 0.97) |
| Greece | 403 (321,503) | 3.07 (2.47,3.80) | 459 (370,570) | 2.88 (2.36,3.53) | -0.21 (-0.26, -0.17) |
| Iceland | 8 (7,10) | 3.08 (2.49,3.78) | 15 (12,18) | 3.20 (2.61,3.93) | 0.13 (0.11, 0.15) |
| Ireland | 61 (50,76) | 1.60 (1.30,1.99) | 124 (99,154) | 1.89 (1.54,2.34) | 0.54 (0.46, 0.62) |
| Israel | 217 (177,263) | 4.50 (3.67,5.51) | 464 (378,564) | 4.39 (3.61,5.32) | -0.07 (-0.10, -0.05) |
| Italy | 2892 (2329,3574) | 3.87 (3.16,4.77) | 3882 (3154,4755) | 3.95 (3.22,4.82) | 0.06 (0.04, 0.09) |
| Luxembourg | 14 (11,17) | 2.92 (2.39,3.53) | 30 (24,37) | 3.45 (2.81,4.21) | 0.55 (0.47, 0.64) |
| Malta | 13 (10,16) | 3.13 (2.52,3.86) | 26 (21,32) | 3.74 (3.06,4.59) | 0.60 (0.51, 0.70) |
| Netherlands | 781 (634,963) | 4.37 (3.56,5.36) | 1192 (951,1473) | 4.66 (3.79,5.76) | 0.21 (0.20, 0.23) |
| Norway | 220 (178,269) | 4.04 (3.31,4.95) | 303 (247,375) | 4.00 (3.29,4.91) | -0.03 (-0.04, -0.02) |
| Portugal | 264 (210,328) | 2.12 (1.71,2.61) | 365 (293,453) | 2.13 (1.74,2.62) | 0.01 (-0.04, 0.06) |
| Spain | 1296 (1044,1589) | 2.73 (2.21,3.34) | 1971 (1573,2448) | 2.82 (2.27,3.46) | 0.10 (0.05, 0.15) |
| Sweden | 693 (560,849) | 6.05 (4.97,7.42) | 931 (756,1153) | 6.30 (5.19,7.70) | 0.14 (0.09, 0.19) |
| Switzerland | 591 (477,728) | 6.76 (5.51,8.31) | 933 (761,1151) | 7.09 (5.80,8.66) | 0.14 (0.09, 0.20) |
| United Kingdom | 1569 (1262,1950) | 2.14 (1.75,2.63) | 2038 (1633,2537) | 2.13 (1.73,2.62) | -0.02 (-0.05, 0.02) |
| Argentina | 813 (656,999) | 2.50 (2.02,3.07) | 1371 (1108,1702) | 2.65 (2.14,3.29) | 0.19 (0.15, 0.23) |
| Chile | 379 (311,466) | 3.25 (2.66,4.00) | 758 (611,931) | 3.30 (2.69,4.03) | 0.03 (-0.01, 0.07) |
| Uruguay | 88 (72,108) | 2.50 (2.04,3.07) | 99 (80,122) | 2.25 (1.83,2.76) | -0.35 (-0.37, -0.32) |
| Canada | 662 (533,810) | 2.15 (1.74,2.63) | 935 (749,1164) | 1.77 (1.43,2.17) | -0.64 (-0.68, -0.59) |
| United States of America | 5322 (4318,6522) | 1.84 (1.50,2.25) | 7690 (6172,9562) | 1.72 (1.39,2.12) | -0.21 (-0.26, -0.16) |
| Antigua and Barbuda | 2 (1,2) | 2.93 (2.39,3.58) | 3 (2,4) | 2.74 (2.24,3.39) | -0.22 (-0.28, -0.16) |
| Bahamas | 5 (4,7) | 2.59 (2.10,3.13) | 9 (7,11) | 2.14 (1.73,2.62) | -0.60 (-0.67, -0.53) |
| Barbados | 7 (6,8) | 2.62 (2.12,3.20) | 10 (8,12) | 2.47 (1.99,3.03) | -0.21 (-0.23, -0.18) |
| Belize | 3 (2,3) | 2.12 (1.71,2.62) | 8 (7,10) | 2.18 (1.76,2.65) | 0.10 (0.01, 0.19) |
| Cuba | 289 (234,352) | 2.68 (2.17,3.27) | 389 (309,482) | 2.55 (2.06,3.14) | -0.17 (-0.21, -0.14) |
| Dominica | 1 (1,2) | 2.12 (1.70,2.59) | 2 (1,2) | 2.23 (1.80,2.72) | 0.16 (0.11, 0.20) |
| Dominican Republic | 150 (122,185) | 2.86 (2.31,3.52) | 286 (231,353) | 2.66 (2.14,3.25) | -0.24 (-0.26, -0.21) |
| Grenada | 2 (1,2) | 2.31 (1.88,2.81) | 2 (2,3) | 2.06 (1.67,2.53) | -0.37 (-0.39, -0.34) |
| Guyana | 12 (10,15) | 2.09 (1.69,2.56) | 14 (12,18) | 1.97 (1.60,2.39) | -0.19 (-0.24, -0.14) |
| Haiti | 88 (72,110) | 1.97 (1.60,2.40) | 189 (152,237) | 1.85 (1.48,2.27) | -0.21 (-0.27, -0.16) |
| Jamaica | 52 (42,63) | 2.63 (2.11,3.22) | 79 (64,98) | 2.62 (2.12,3.23) | -0.02 (-0.04, 0.01) |
| Saint Lucia | 2 (2,3) | 2.28 (1.85,2.79) | 5 (4,6) | 2.33 (1.88,2.87) | 0.08 (0.04, 0.12) |
| Saint Vincent and the Grenadines | 2 (1,2) | 2.11 (1.71,2.55) | 3 (2,3) | 2.15 (1.74,2.66) | 0.07 (-0.00, 0.14) |
| Suriname | 8 (7,10) | 2.68 (2.18,3.29) | 16 (13,19) | 2.51 (2.05,3.10) | -0.20 (-0.25, -0.15) |
| Trinidad and Tobago | 22 (18,26) | 2.12 (1.71,2.57) | 39 (31,48) | 2.27 (1.83,2.79) | 0.22 (0.13, 0.30) |
| Bolivia (Plurinational State of) | 119 (96,146) | 2.61 (2.11,3.17) | 266 (215,329) | 2.45 (1.98,3.00) | -0.20 (-0.26, -0.14) |
| Ecuador | 213 (174,264) | 2.92 (2.34,3.58) | 519 (422,640) | 2.97 (2.41,3.63) | 0.05 (-0.00, 0.11) |
| Peru | 481 (394,592) | 3.00 (2.42,3.70) | 993 (804,1224) | 2.77 (2.23,3.39) | -0.26 (-0.29, -0.23) |
| Colombia | 731 (600,887) | 2.96 (2.42,3.61) | 1817 (1474,2241) | 3.38 (2.76,4.15) | 0.43 (0.38, 0.47) |
| Costa Rica | 69 (56,85) | 2.90 (2.35,3.58) | 189 (153,233) | 3.58 (2.90,4.38) | 0.68 (0.61, 0.75) |
| El Salvador | 96 (79,117) | 2.47 (1.99,3.01) | 227 (186,281) | 3.62 (2.95,4.48) | 1.22 (1.16, 1.29) |
| Guatemala | 123 (101,153) | 2.30 (1.86,2.82) | 393 (319,486) | 2.94 (2.37,3.61) | 0.80 (0.73, 0.87) |
| Honduras | 73 (60,90) | 2.35 (1.91,2.88) | 246 (201,304) | 2.96 (2.40,3.65) | 0.75 (0.67, 0.83) |
| Mexico | 1633 (1345,2012) | 2.61 (2.12,3.18) | 4287 (3483,5273) | 3.22 (2.63,3.95) | 0.67 (0.60, 0.75) |
| Nicaragua | 59 (48,73) | 2.35 (1.91,2.90) | 175 (142,214) | 2.94 (2.38,3.58) | 0.68 (0.47, 0.90) |
| Panama | 54 (44,65) | 2.81 (2.28,3.44) | 152 (123,188) | 3.47 (2.80,4.27) | 0.68 (0.62, 0.75) |
| Venezuela (Bolivarian Republic of) | 424 (344,518) | 3.04 (2.45,3.69) | 935 (758,1156) | 3.20 (2.60,3.95) | 0.17 (0.03, 0.32) |
| Brazil | 2811 (2296,3451) | 2.34 (1.90,2.87) | 6078 (4890,7487) | 2.48 (2.01,3.04) | 0.19 (0.10, 0.27) |
| Paraguay | 74 (61,91) | 2.51 (2.02,3.10) | 161 (130,196) | 2.40 (1.95,2.93) | -0.12 (-0.21, -0.03) |
| Algeria | 308 (251,380) | 1.72 (1.39,2.14) | 782 (631,979) | 1.86 (1.49,2.31) | 0.23 (0.18, 0.27) |
| Bahrain | 7 (6,9) | 1.85 (1.50,2.24) | 28 (23,36) | 1.83 (1.48,2.25) | -0.06 (-0.11, -0.01) |
| Egypt | 1120 (915,1402) | 2.71 (2.22,3.33) | 1547 (1262,1927) | 1.74 (1.41,2.14) | -1.45 (-1.59, -1.32) |
| Iran (Islamic Republic of) | 807 (660,992) | 2.07 (1.69,2.54) | 1904 (1533,2361) | 2.13 (1.73,2.62) | 0.04 (-0.13, 0.22) |
| Iraq | 211 (173,261) | 1.71 (1.40,2.10) | 640 (525,798) | 1.87 (1.52,2.31) | 0.28 (0.19, 0.37) |
| Jordan | 53 (43,66) | 2.21 (1.79,2.71) | 243 (197,299) | 2.25 (1.82,2.78) | 0.07 (0.00, 0.15) |
| Kuwait | 27 (22,34) | 2.06 (1.66,2.54) | 106 (83,135) | 2.17 (1.75,2.64) | 0.16 (0.11, 0.20) |
| Lebanon | 53 (43,65) | 2.05 (1.67,2.53) | 123 (99,153) | 2.08 (1.68,2.58) | 0.02 (-0.05, 0.08) |
| Libya | 64 (52,79) | 2.21 (1.78,2.71) | 144 (115,181) | 2.11 (1.70,2.58) | -0.15 (-0.22, -0.09) |
| Morocco | 344 (282,426) | 1.78 (1.45,2.17) | 678 (546,846) | 1.81 (1.46,2.22) | 0.04 (-0.01, 0.09) |
| Palestine | 28 (23,34) | 2.16 (1.76,2.66) | 75 (61,93) | 1.87 (1.52,2.28) | -0.44 (-0.54, -0.34) |
| Oman | 27 (22,34) | 1.99 (1.63,2.43) | 83 (67,108) | 2.02 (1.64,2.47) | -0.01 (-0.09, 0.08) |
| Qatar | 7 (5,9) | 1.86 (1.49,2.28) | 54 (42,70) | 1.89 (1.54,2.30) | 0.04 (-0.01, 0.10) |
| Saudi Arabia | 179 (144,224) | 1.62 (1.32,1.99) | 673 (535,842) | 1.91 (1.55,2.32) | 0.55 (0.41, 0.69) |
| Syrian Arab Republic | 156 (127,194) | 1.87 (1.52,2.31) | 280 (227,346) | 1.96 (1.60,2.41) | 0.17 (0.03, 0.31) |
| Tunisia | 142 (116,174) | 2.15 (1.75,2.64) | 306 (246,380) | 2.32 (1.88,2.86) | 0.24 (0.17, 0.31) |
| Turkey | 858 (704,1076) | 1.86 (1.50,2.32) | 2423 (1942,3033) | 2.60 (2.10,3.23) | 1.08 (0.97, 1.19) |
| United Arab Emirates | 24 (19,32) | 1.76 (1.44,2.17) | 175 (134,230) | 1.67 (1.36,2.04) | -0.15 (-0.23, -0.07) |
| Yemen | 203 (167,249) | 2.50 (2.03,3.03) | 505 (413,634) | 2.15 (1.73,2.64) | -0.52 (-0.62, -0.42) |
| Afghanistan | 110 (90,134) | 1.43 (1.16,1.76) | 278 (225,344) | 1.46 (1.18,1.78) | 0.05 (-0.01, 0.11) |
| Bangladesh | 918 (750,1147) | 1.28 (1.02,1.59) | 2842 (2309,3541) | 1.81 (1.46,2.23) | 1.12 (1.02, 1.21) |
| Bhutan | 8 (6,10) | 1.90 (1.55,2.32) | 12 (10,15) | 1.64 (1.33,2.02) | -0.48 (-0.55, -0.40) |
| India | 10592 (8715,13169) | 1.62 (1.32,1.98) | 23742 (19176,29559) | 1.74 (1.41,2.12) | 0.22 (0.20, 0.23) |
| Nepal | 204 (166,254) | 1.49 (1.20,1.84) | 445 (362,545) | 1.59 (1.29,1.94) | 0.21 (0.19, 0.23) |
| Pakistan | 1174 (958,1433) | 1.51 (1.22,1.83) | 2520 (2036,3133) | 1.38 (1.11,1.70) | -0.26 (-0.39, -0.13) |
| Angola | 121 (97,150) | 1.86 (1.48,2.25) | 331 (269,408) | 1.61 (1.31,1.97) | -0.48 (-0.54, -0.42) |
| Central African Republic | 33 (26,40) | 1.85 (1.49,2.26) | 61 (49,76) | 1.67 (1.35,2.03) | -0.32 (-0.35, -0.29) |
| Congo | 41 (33,50) | 2.57 (2.09,3.12) | 88 (71,108) | 2.13 (1.72,2.62) | -0.59 (-0.76, -0.42) |
| Democratic Republic of the Congo | 751 (616,934) | 3.13 (2.53,3.85) | 1166 (944,1452) | 1.93 (1.55,2.33) | -1.57 (-1.68, -1.46) |
| Equatorial Guinea | 5 (4,6) | 1.86 (1.50,2.25) | 18 (15,23) | 1.86 (1.49,2.30) | 0.03 (-0.05, 0.11) |
| Gabon | 21 (17,25) | 2.84 (2.30,3.43) | 30 (24,37) | 2.05 (1.66,2.51) | -1.06 (-1.12, -1.00) |
| Burundi | 89 (72,110) | 2.52 (2.01,3.07) | 146 (116,182) | 1.70 (1.36,2.08) | -1.29 (-1.40, -1.18) |
| Comoros | 6 (5,7) | 2.00 (1.59,2.42) | 11 (9,13) | 1.70 (1.37,2.09) | -0.56 (-0.70, -0.42) |
| Djibouti | 5 (4,6) | 1.83 (1.46,2.24) | 19 (16,24) | 1.92 (1.57,2.35) | 0.12 (0.04, 0.21) |
| Eritrea | 45 (37,56) | 2.19 (1.81,2.64) | 83 (66,104) | 1.77 (1.42,2.15) | -0.71 (-0.80, -0.62) |
| Ethiopia | 787 (648,972) | 2.48 (2.02,3.01) | 1857 (1521,2307) | 2.53 (2.06,3.09) | 0.07 (-0.06, 0.19) |
| Kenya | 350 (286,432) | 2.49 (2.02,3.02) | 927 (762,1139) | 2.59 (2.10,3.15) | 0.14 (0.01, 0.27) |
| Madagascar | 177 (145,220) | 2.29 (1.88,2.82) | 380 (304,479) | 1.96 (1.58,2.39) | -0.51 (-0.59, -0.44) |
| Malawi | 131 (105,160) | 2.14 (1.71,2.61) | 203 (163,253) | 1.60 (1.29,1.95) | -0.95 (-1.02, -0.89) |
| Mauritius | 32 (26,39) | 3.27 (2.67,4.03) | 52 (42,64) | 3.24 (2.65,3.96) | -0.03 (-0.04, -0.01) |
| Mozambique | 178 (145,219) | 2.03 (1.63,2.48) | 331 (268,412) | 1.73 (1.40,2.13) | -0.50 (-0.54, -0.46) |
| Rwanda | 120 (97,149) | 2.68 (2.15,3.27) | 165 (133,206) | 1.69 (1.36,2.11) | -1.49 (-1.58, -1.41) |
| Seychelles | 1 (1,1) | 1.80 (1.45,2.19) | 2 (2,3) | 1.85 (1.50,2.25) | 0.09 (0.06, 0.12) |
| Somalia | 96 (77,120) | 2.07 (1.65,2.52) | 215 (171,271) | 1.73 (1.39,2.12) | -0.58 (-0.62, -0.55) |
| United Republic of Tanzania | 387 (318,471) | 2.33 (1.90,2.86) | 715 (576,882) | 1.79 (1.44,2.17) | -0.85 (-0.93, -0.77) |
| Uganda | 321 (256,400) | 3.04 (2.43,3.75) | 530 (432,654) | 2.00 (1.60,2.41) | -1.49 (-2.10, -0.87) |
| Zambia | 105 (86,130) | 2.19 (1.77,2.69) | 203 (165,252) | 1.60 (1.29,1.96) | -1.03 (-1.08, -0.98) |
| Botswana | 20 (16,24) | 2.26 (1.83,2.72) | 39 (32,49) | 1.95 (1.57,2.40) | -0.48 (-0.50, -0.46) |
| Lesotho | 18 (15,22) | 1.69 (1.37,2.07) | 25 (20,30) | 1.70 (1.38,2.07) | 0.01 (-0.01, 0.03) |
| Namibia | 26 (21,32) | 2.75 (2.21,3.37) | 51 (42,62) | 2.67 (2.18,3.22) | -0.10 (-0.18, -0.03) |
| South Africa | 605 (499,737) | 2.19 (1.78,2.66) | 1267 (1021,1544) | 2.38 (1.93,2.88) | 0.24 (0.19, 0.30) |
| Eswatini | 8 (7,10) | 1.72 (1.39,2.10) | 15 (12,18) | 1.74 (1.38,2.12) | 0.04 (0.03, 0.05) |
| Zimbabwe | 118 (96,145) | 1.79 (1.45,2.18) | 200 (162,250) | 1.85 (1.50,2.29) | 0.07 (-0.04, 0.18) |
| Benin | 80 (66,98) | 2.65 (2.14,3.27) | 216 (176,267) | 2.54 (2.05,3.10) | -0.11 (-0.26, 0.03) |
| Burkina Faso | 131 (107,161) | 2.15 (1.73,2.64) | 303 (250,371) | 2.10 (1.71,2.57) | -0.07 (-0.14, 0.01) |
| Cameroon | 164 (135,200) | 2.42 (1.96,2.92) | 492 (403,607) | 2.35 (1.90,2.89) | -0.10 (-0.18, -0.02) |
| Cabo Verde | 6 (5,7) | 2.17 (1.77,2.67) | 14 (11,17) | 2.61 (2.11,3.17) | 0.60 (0.49, 0.70) |
| Chad | 70 (57,85) | 1.79 (1.44,2.20) | 214 (174,268) | 2.13 (1.71,2.63) | 0.55 (0.48, 0.62) |
| Cote d'Ivoire | 173 (141,214) | 2.31 (1.86,2.81) | 377 (305,469) | 2.01 (1.64,2.45) | -0.46 (-0.53, -0.38) |
| Gambia | 13 (11,17) | 2.21 (1.79,2.70) | 33 (27,41) | 2.03 (1.65,2.50) | -0.23 (-0.41, -0.05) |
| Ghana | 340 (279,413) | 3.48 (2.81,4.25) | 807 (666,1000) | 3.26 (2.65,4.00) | -0.19 (-0.41, 0.03) |
| Guinea | 88 (71,107) | 2.07 (1.68,2.53) | 195 (159,239) | 2.25 (1.83,2.71) | 0.26 (0.16, 0.37) |
| Guinea-Bissau | 11 (9,14) | 1.81 (1.46,2.20) | 27 (22,34) | 2.07 (1.68,2.53) | 0.42 (0.33, 0.50) |
| Liberia | 51 (42,63) | 3.09 (2.53,3.79) | 82 (67,103) | 2.21 (1.79,2.73) | -1.05 (-1.22, -0.88) |
| Mali | 123 (101,150) | 2.16 (1.75,2.60) | 314 (257,388) | 2.17 (1.75,2.65) | -0.00 (-0.09, 0.08) |
| Mauritania | 25 (20,31) | 1.76 (1.41,2.18) | 75 (61,93) | 2.45 (1.98,3.02) | 1.03 (0.81, 1.26) |
| Niger | 90 (73,112) | 1.90 (1.52,2.33) | 302 (247,375) | 2.16 (1.74,2.65) | 0.40 (0.34, 0.46) |
| Nigeria | 1483 (1219,1827) | 2.39 (1.93,2.92) | 5344 (4376,6636) | 3.66 (2.95,4.50) | 1.37 (1.28, 1.46) |
| Sao Tome and Principe | 2 (1,2) | 2.01 (1.63,2.49) | 4 (3,5) | 2.44 (1.98,3.01) | 0.63 (0.59, 0.67) |
| Senegal | 150 (124,186) | 3.06 (2.48,3.73) | 268 (221,332) | 2.37 (1.92,2.90) | -0.87 (-1.10, -0.64) |
| Sierra Leone | 58 (47,71) | 2.01 (1.61,2.44) | 131 (106,163) | 2.18 (1.76,2.71) | 0.26 (0.12, 0.40) |
| Togo | 56 (46,69) | 2.59 (2.09,3.15) | 121 (98,150) | 2.05 (1.66,2.51) | -0.78 (-0.87, -0.68) |
| American Samoa | 1 (1,1) | 2.13 (1.73,2.63) | 1 (1,1) | 2.01 (1.62,2.46) | -0.16 (-0.22, -0.11) |
| Bermuda | 2 (1,2) | 2.89 (2.34,3.56) | 3 (2,3) | 2.73 (2.22,3.32) | -0.20 (-0.23, -0.16) |
| Cook Islands | 0 (0,0) | 2.29 (1.87,2.77) | 0 (0,1) | 2.17 (1.77,2.68) | -0.16 (-0.22, -0.10) |
| Greenland | 1 (1,1) | 1.60 (1.30,1.95) | 1 (1,1) | 1.32 (1.06,1.62) | -0.63 (-0.66, -0.61) |
| Guam | 2 (2,3) | 2.20 (1.77,2.67) | 4 (3,5) | 2.06 (1.69,2.51) | -0.19 (-0.25, -0.13) |
| Monaco | 2 (1,2) | 3.37 (2.75,4.16) | 2 (2,3) | 3.66 (3.00,4.47) | 0.27 (0.24, 0.29) |
| Nauru | 0 (0,0) | 1.71 (1.38,2.09) | 0 (0,0) | 1.63 (1.32,1.98) | -0.14 (-0.19, -0.09) |
| Niue | 0 (0,0) | 2.19 (1.78,2.66) | 0 (0,0) | 1.99 (1.62,2.44) | -0.30 (-0.35, -0.24) |
| Northern Mariana Islands | 1 (1,1) | 2.24 (1.81,2.74) | 1 (1,1) | 2.11 (1.72,2.56) | -0.18 (-0.23, -0.12) |
| Palau | 0 (0,0) | 2.05 (1.65,2.49) | 0 (0,1) | 1.84 (1.50,2.24) | -0.32 (-0.38, -0.26) |
| Puerto Rico | 100 (80,122) | 2.76 (2.20,3.36) | 126 (101,158) | 2.62 (2.10,3.18) | -0.18 (-0.21, -0.15) |
| Saint Kitts and Nevis | 1 (1,1) | 2.34 (1.90,2.84) | 1 (1,2) | 1.97 (1.60,2.41) | -0.55 (-0.59, -0.52) |
| San Marino | 1 (1,1) | 3.58 (2.91,4.42) | 2 (2,2) | 3.85 (3.14,4.70) | 0.24 (0.22, 0.25) |
| Tokelau | 0 (0,0) | 1.87 (1.53,2.29) | 0 (0,0) | 1.95 (1.58,2.39) | 0.17 (0.08, 0.27) |
| Tuvalu | 0 (0,0) | 1.95 (1.58,2.41) | 0 (0,0) | 1.69 (1.36,2.09) | -0.44 (-0.50, -0.38) |
| United States Virgin Islands | 3 (2,3) | 2.58 (2.08,3.15) | 3 (2,4) | 2.44 (1.97,2.95) | -0.19 (-0.21, -0.17) |
| South Sudan | 77 (62,95) | 1.98 (1.58,2.48) | 118 (95,147) | 1.85 (1.49,2.26) | -0.24 (-0.36, -0.11) |
| Sudan | 237 (194,293) | 1.74 (1.40,2.15) | 531 (439,667) | 1.68 (1.37,2.05) | -0.12 (-0.16, -0.08) |

Note: AAPC, average annual percentage change; CI, confidence interval; UI, uncertainty interval.

**Supplementary Table S6** Age-standardized mortality rate and AAPC of PAH in 204 countries and territories, 1990-2021.

|  | Mortality | | | | |
| --- | --- | --- | --- | --- | --- |
|  | Cases in 1990 | Age-standardized rate in 1990 (per 100,000) | Cases in 2021 | Age-standardized rate in 2021 (per 100,000) | 1990–2021 AAPC (95% CI) |
| China | 4059 (3099,5452) | 0.61 (0.46,0.83) | 7318 (4836,9076) | 0.42 (0.28,0.51) | -1.26 (-1.53, -0.99) |
| Democratic People's Republic of Korea | 43 (29,78) | 0.34 (0.22,0.65) | 95 (67,160) | 0.35 (0.24,0.58) | 0.07 (-0.01, 0.15) |
| Taiwan (Province of China) | 13 (12,13) | 0.10 (0.09,0.10) | 77 (66,85) | 0.18 (0.16,0.20) | 1.99 (0.97, 3.02) |
| Cambodia | 12 (6,26) | 0.16 (0.08,0.43) | 17 (10,38) | 0.13 (0.07,0.35) | -0.74 (-0.82, -0.66) |
| Indonesia | 185 (106,426) | 0.14 (0.08,0.42) | 268 (170,672) | 0.12 (0.07,0.37) | -0.52 (-0.59, -0.45) |
| Lao People's Democratic Republic | 8 (3,19) | 0.23 (0.11,0.54) | 9 (5,20) | 0.15 (0.09,0.36) | -1.30 (-1.39, -1.21) |
| Malaysia | 10 (7,30) | 0.08 (0.05,0.30) | 19 (11,65) | 0.07 (0.04,0.26) | -0.65 (-1.15, -0.14) |
| Maldives | 1 (1,1) | 0.52 (0.37,0.69) | 1 (1,1) | 0.19 (0.15,0.30) | -3.25 (-3.38, -3.11) |
| Myanmar | 70 (37,151) | 0.22 (0.12,0.55) | 75 (48,178) | 0.15 (0.10,0.42) | -1.14 (-1.22, -1.06) |
| Philippines | 52 (34,115) | 0.11 (0.06,0.33) | 70 (48,184) | 0.08 (0.05,0.24) | -1.13 (-1.40, -0.85) |
| Sri Lanka | 55 (39,92) | 0.42 (0.32,0.70) | 101 (63,148) | 0.42 (0.27,0.60) | 0.11 (-0.31, 0.54) |
| Thailand | 40 (25,123) | 0.09 (0.06,0.38) | 62 (35,282) | 0.07 (0.04,0.28) | -0.94 (-1.28, -0.59) |
| Timor-Leste | 1 (1,3) | 0.19 (0.09,0.49) | 2 (1,4) | 0.15 (0.08,0.43) | -0.68 (-0.90, -0.47) |
| Viet Nam | 70 (38,194) | 0.15 (0.07,0.50) | 106 (53,332) | 0.12 (0.06,0.40) | -0.78 (-0.82, -0.74) |
| Fiji | 1 (1,2) | 0.21 (0.13,0.44) | 1 (1,2) | 0.15 (0.08,0.32) | -1.05 (-1.13, -0.97) |
| Kiribati | 0 (0,0) | 0.30 (0.17,0.68) | 0 (0,0) | 0.25 (0.16,0.57) | -0.57 (-0.62, -0.52) |
| Marshall Islands | 0 (0,0) | 0.33 (0.20,0.69) | 0 (0,0) | 0.23 (0.14,0.50) | -1.14 (-1.19, -1.08) |
| Micronesia (Federated States of) | 0 (0,0) | 0.36 (0.22,0.80) | 0 (0,0) | 0.24 (0.15,0.55) | -1.30 (-1.34, -1.25) |
| Papua New Guinea | 9 (5,14) | 0.30 (0.18,0.58) | 20 (13,36) | 0.27 (0.17,0.55) | -0.31 (-0.42, -0.21) |
| Samoa | 0 (0,1) | 0.27 (0.18,0.61) | 0 (0,1) | 0.20 (0.13,0.47) | -1.00 (-1.05, -0.96) |
| Solomon Islands | 0 (0,1) | 0.26 (0.14,0.54) | 1 (0,1) | 0.22 (0.13,0.51) | -0.59 (-0.70, -0.48) |
| Tonga | 0 (0,0) | 0.20 (0.13,0.45) | 0 (0,0) | 0.14 (0.09,0.34) | -1.10 (-1.28, -0.92) |
| Vanuatu | 0 (0,0) | 0.28 (0.17,0.57) | 0 (0,1) | 0.22 (0.14,0.52) | -0.70 (-0.87, -0.53) |
| Armenia | 7 (6,8) | 0.25 (0.21,0.31) | 3 (3,4) | 0.08 (0.06,0.09) | -3.83 (-4.38, -3.28) |
| Azerbaijan | 37 (25,52) | 0.70 (0.48,0.99) | 54 (32,83) | 0.57 (0.36,0.85) | -0.69 (-0.99, -0.39) |
| Georgia | 21 (16,27) | 0.37 (0.29,0.47) | 58 (45,73) | 1.01 (0.79,1.27) | 3.21 (2.45, 3.98) |
| Kazakhstan | 6 (5,7) | 0.05 (0.03,0.06) | 7 (6,9) | 0.05 (0.04,0.06) | 0.08 (-0.24, 0.40) |
| Kyrgyzstan | 3 (3,4) | 0.09 (0.08,0.11) | 2 (2,3) | 0.05 (0.04,0.06) | -2.16 (-2.89, -1.43) |
| Mongolia | 20 (11,32) | 1.73 (0.99,2.86) | 34 (20,44) | 1.59 (0.91,2.05) | -0.35 (-0.67, -0.02) |
| Tajikistan | 41 (25,54) | 1.21 (0.74,1.66) | 53 (34,73) | 0.81 (0.53,1.09) | -1.34 (-1.76, -0.91) |
| Turkmenistan | 4 (3,5) | 0.18 (0.13,0.24) | 9 (7,12) | 0.23 (0.17,0.31) | 0.75 (0.46, 1.04) |
| Uzbekistan | 68 (54,85) | 0.46 (0.34,0.61) | 97 (77,119) | 0.37 (0.30,0.46) | -0.84 (-1.64, -0.04) |
| Albania | 11 (8,15) | 0.52 (0.36,0.70) | 11 (7,19) | 0.28 (0.19,0.48) | -1.98 (-2.42, -1.54) |
| Bosnia and Herzegovina | 10 (7,14) | 0.27 (0.19,0.37) | 12 (9,16) | 0.21 (0.15,0.27) | -0.84 (-1.12, -0.55) |
| Bulgaria | 16 (13,18) | 0.16 (0.13,0.18) | 11 (8,15) | 0.09 (0.07,0.12) | -1.70 (-2.60, -0.80) |
| Croatia | 6 (6,7) | 0.12 (0.10,0.14) | 4 (3,5) | 0.05 (0.04,0.05) | -3.10 (-3.71, -2.48) |
| Czechia | 51 (43,60) | 0.39 (0.33,0.46) | 88 (76,101) | 0.43 (0.36,0.49) | 0.44 (-0.26, 1.14) |
| Hungary | 43 (38,49) | 0.32 (0.28,0.37) | 31 (25,38) | 0.16 (0.13,0.21) | -1.99 (-2.82, -1.16) |
| North Macedonia | 3 (2,4) | 0.15 (0.10,0.26) | 3 (2,6) | 0.12 (0.07,0.23) | -0.69 (-1.19, -0.18) |
| Montenegro | 0 (0,1) | 0.04 (0.02,0.24) | 0 (0,2) | 0.03 (0.02,0.20) | -0.77 (-1.07, -0.47) |
| Poland | 69 (60,79) | 0.17 (0.14,0.19) | 77 (69,84) | 0.12 (0.11,0.13) | -1.14 (-1.48, -0.81) |
| Romania | 120 (96,144) | 0.48 (0.39,0.57) | 170 (146,195) | 0.49 (0.42,0.55) | -0.04 (-0.39, 0.31) |
| Serbia | 13 (8,32) | 0.14 (0.09,0.39) | 15 (9,30) | 0.09 (0.06,0.19) | -1.37 (-1.60, -1.13) |
| Slovakia | 6 (4,12) | 0.10 (0.06,0.23) | 6 (3,11) | 0.06 (0.04,0.13) | -1.55 (-1.79, -1.31) |
| Slovenia | 1 (1,1) | 0.04 (0.03,0.04) | 2 (1,2) | 0.04 (0.03,0.04) | -0.12 (-0.79, 0.56) |
| Belarus | 5 (4,6) | 0.04 (0.03,0.05) | 6 (5,7) | 0.04 (0.03,0.05) | -0.35 (-0.54, -0.16) |
| Estonia | 0 (0,0) | 0.02 (0.02,0.03) | 1 (1,1) | 0.04 (0.03,0.04) | 1.67 (-0.19, 3.55) |
| Latvia | 1 (1,1) | 0.02 (0.02,0.03) | 4 (4,5) | 0.10 (0.09,0.12) | 4.71 (3.42, 6.01) |
| Lithuania | 1 (1,2) | 0.04 (0.03,0.04) | 4 (4,5) | 0.08 (0.07,0.09) | 2.58 (0.61, 4.58) |
| Republic of Moldova | 0.00 (0.00,0.00) | 0.00 (0.00,0.00) | 0.00 (0.00,0.00) | 0.01 (0.01,0.01) | 3.96 (2.83, 5.10) |
| Russian Federation | 515 (465,600) | 0.34 (0.31,0.39) | 221 (204,238) | 0.10 (0.10,0.11) | -3.70 (-4.43, -2.97) |
| Ukraine | 41 (37,46) | 0.07 (0.06,0.07) | 42 (31,53) | 0.07 (0.05,0.09) | -0.01 (-1.23, 1.24) |
| Brunei Darussalam | 1 (0,1) | 0.48 (0.29,0.65) | 1 (1,1) | 0.27 (0.20,0.34) | -1.88 (-2.12, -1.63) |
| Japan | 385 (366,401) | 0.29 (0.28,0.30) | 1002 (786,1147) | 0.29 (0.25,0.32) | -0.05 (-0.37, 0.27) |
| Republic of Korea | 42 (29,72) | 0.13 (0.09,0.27) | 39 (22,136) | 0.06 (0.03,0.17) | -2.67 (-2.89, -2.45) |
| Singapore | 6 (5,6) | 0.23 (0.21,0.24) | 6 (5,7) | 0.09 (0.08,0.10) | -3.01 (-3.83, -2.17) |
| Australia | 40 (34,52) | 0.22 (0.19,0.29) | 50 (42,57) | 0.12 (0.10,0.13) | -2.01 (-2.45, -1.57) |
| New Zealand | 5 (5,6) | 0.14 (0.13,0.15) | 8 (7,8) | 0.10 (0.09,0.11) | -0.93 (-2.08, 0.25) |
| Andorra | 0 (0,0) | 0.25 (0.18,0.35) | 0 (0,0) | 0.11 (0.07,0.17) | -2.57 (-2.82, -2.33) |
| Austria | 20 (18,21) | 0.18 (0.16,0.19) | 25 (22,28) | 0.13 (0.12,0.14) | -1.05 (-1.53, -0.56) |
| Belgium | 44 (38,56) | 0.31 (0.27,0.39) | 45 (38,50) | 0.18 (0.16,0.20) | -1.79 (-1.91, -1.66) |
| Cyprus | 13 (7,17) | 2.09 (1.09,2.75) | 15 (7,19) | 0.80 (0.38,1.02) | -3.00 (-3.53, -2.48) |
| Denmark | 17 (15,18) | 0.23 (0.21,0.25) | 21 (18,23) | 0.18 (0.15,0.19) | -0.88 (-1.32, -0.43) |
| Finland | 6 (5,7) | 0.09 (0.08,0.10) | 8 (7,9) | 0.06 (0.05,0.07) | -1.11 (-1.51, -0.71) |
| France | 239 (191,296) | 0.30 (0.24,0.37) | 325 (279,361) | 0.21 (0.19,0.23) | -1.15 (-1.36, -0.94) |
| Germany | 304 (254,350) | 0.27 (0.23,0.31) | 544 (453,604) | 0.26 (0.22,0.28) | -0.24 (-0.72, 0.24) |
| Greece | 32 (29,34) | 0.24 (0.23,0.26) | 87 (76,98) | 0.32 (0.29,0.36) | 0.92 (0.57, 1.27) |
| Iceland | 0 (0,0) | 0.15 (0.14,0.17) | 1 (1,1) | 0.13 (0.11,0.15) | -0.64 (-1.06, -0.21) |
| Ireland | 6 (5,6) | 0.15 (0.14,0.16) | 8 (7,9) | 0.11 (0.10,0.12) | -1.10 (-1.65, -0.54) |
| Israel | 22 (19,29) | 0.48 (0.41,0.61) | 35 (30,39) | 0.27 (0.23,0.30) | -1.80 (-1.96, -1.63) |
| Italy | 171 (159,179) | 0.21 (0.20,0.23) | 138 (115,153) | 0.09 (0.08,0.10) | -2.84 (-3.12, -2.55) |
| Luxembourg | 1 (1,1) | 0.27 (0.25,0.29) | 2 (2,3) | 0.21 (0.18,0.23) | -0.87 (-1.15, -0.59) |
| Malta | 0 (0,0) | 0.09 (0.08,0.10) | 1 (1,1) | 0.08 (0.07,0.10) | -0.38 (-0.77, 0.01) |
| Netherlands | 30 (28,33) | 0.16 (0.15,0.17) | 48 (41,53) | 0.14 (0.12,0.15) | -0.53 (-0.90, -0.16) |
| Norway | 7 (7,8) | 0.14 (0.13,0.15) | 5 (4,5) | 0.05 (0.05,0.05) | -3.59 (-5.64, -1.49) |
| Portugal | 28 (26,30) | 0.25 (0.23,0.26) | 56 (48,63) | 0.22 (0.19,0.24) | -0.56 (-1.20, 0.07) |
| Spain | 131 (121,140) | 0.27 (0.25,0.29) | 241 (200,270) | 0.22 (0.19,0.24) | -0.67 (-1.00, -0.33) |
| Sweden | 17 (16,19) | 0.14 (0.13,0.15) | 26 (22,29) | 0.12 (0.11,0.14) | -0.52 (-1.49, 0.46) |
| Switzerland | 35 (30,43) | 0.36 (0.31,0.44) | 37 (31,42) | 0.19 (0.16,0.21) | -2.11 (-2.46, -1.76) |
| United Kingdom | 106 (96,132) | 0.14 (0.13,0.18) | 119 (107,126) | 0.10 (0.10,0.11) | -1.10 (-1.37, -0.84) |
| Argentina | 137 (120,153) | 0.43 (0.38,0.48) | 108 (100,118) | 0.20 (0.19,0.22) | -2.48 (-2.71, -2.26) |
| Chile | 24 (23,26) | 0.22 (0.20,0.23) | 34 (30,37) | 0.14 (0.13,0.16) | -1.33 (-1.59, -1.07) |
| Uruguay | 8 (7,9) | 0.23 (0.20,0.25) | 8 (7,9) | 0.16 (0.14,0.17) | -1.30 (-1.49, -1.11) |
| Canada | 93 (85,100) | 0.31 (0.29,0.33) | 95 (83,104) | 0.14 (0.13,0.15) | -2.30 (-2.82, -1.78) |
| United States of America | 971 (855,1070) | 0.32 (0.28,0.35) | 1785 (1535,1945) | 0.31 (0.27,0.33) | -0.09 (-0.30, 0.13) |
| Antigua and Barbuda | 0 (0,0) | 0.11 (0.10,0.12) | 0 (0,0) | 0.04 (0.04,0.04) | -3.20 (-4.02, -2.38) |
| Bahamas | 2 (2,2) | 1.06 (0.93,1.18) | 2 (1,2) | 0.40 (0.32,0.50) | -3.02 (-3.41, -2.64) |
| Barbados | 3 (3,3) | 1.06 (0.91,1.19) | 2 (1,2) | 0.36 (0.29,0.45) | -3.16 (-3.56, -2.76) |
| Belize | 1 (1,1) | 0.46 (0.42,0.52) | 0 (0,1) | 0.15 (0.13,0.16) | -3.68 (-4.24, -3.13) |
| Cuba | 11 (10,12) | 0.11 (0.10,0.12) | 9 (8,10) | 0.05 (0.04,0.06) | -2.14 (-2.44, -1.84) |
| Dominica | 0 (0,0) | 0.18 (0.11,0.32) | 0 (0,0) | 0.09 (0.07,0.23) | -2.01 (-2.19, -1.83) |
| Dominican Republic | 12 (9,16) | 0.17 (0.13,0.32) | 9 (6,23) | 0.08 (0.06,0.23) | -2.17 (-2.50, -1.83) |
| Grenada | 1 (1,1) | 0.81 (0.68,0.98) | 0 (0,0) | 0.26 (0.22,0.29) | -3.56 (-4.13, -2.98) |
| Guyana | 1 (1,1) | 0.17 (0.15,0.19) | 3 (2,3) | 0.39 (0.30,0.50) | 2.88 (2.38, 3.38) |
| Haiti | 55 (21,95) | 0.87 (0.43,1.27) | 53 (27,87) | 0.49 (0.28,0.73) | -1.79 (-1.88, -1.71) |
| Jamaica | 4 (4,5) | 0.20 (0.18,0.23) | 2 (2,3) | 0.07 (0.05,0.09) | -3.30 (-4.06, -2.53) |
| Saint Lucia | 1 (1,1) | 0.80 (0.72,0.89) | 1 (0,1) | 0.24 (0.20,0.29) | -3.81 (-4.31, -3.30) |
| Saint Vincent and the Grenadines | 0 (0,0) | 0.09 (0.08,0.10) | 0 (0,0) | 0.06 (0.05,0.07) | -1.12 (-1.57, -0.68) |
| Suriname | 2 (1,3) | 0.74 (0.49,0.90) | 2 (1,3) | 0.32 (0.24,0.47) | -2.61 (-2.93, -2.28) |
| Trinidad and Tobago | 5 (4,5) | 0.47 (0.44,0.52) | 3 (2,4) | 0.18 (0.14,0.24) | -2.77 (-3.14, -2.40) |
| Bolivia (Plurinational State of) | 23 (12,37) | 0.42 (0.26,0.62) | 21 (15,30) | 0.24 (0.17,0.34) | -1.81 (-1.87, -1.75) |
| Ecuador | 18 (14,22) | 0.27 (0.20,0.34) | 27 (23,32) | 0.18 (0.15,0.21) | -1.38 (-1.73, -1.02) |
| Peru | 42 (29,57) | 0.25 (0.19,0.32) | 43 (31,63) | 0.13 (0.09,0.18) | -2.06 (-2.92, -1.20) |
| Colombia | 34 (28,43) | 0.15 (0.12,0.19) | 52 (43,63) | 0.10 (0.08,0.12) | -1.44 (-1.75, -1.13) |
| Costa Rica | 8 (8,9) | 0.43 (0.39,0.48) | 6 (5,6) | 0.10 (0.09,0.12) | -4.59 (-5.15, -4.02) |
| El Salvador | 12 (9,16) | 0.29 (0.20,0.38) | 8 (5,16) | 0.12 (0.08,0.24) | -2.87 (-3.08, -2.66) |
| Guatemala | 14 (12,16) | 0.26 (0.22,0.30) | 7 (6,8) | 0.06 (0.05,0.07) | -4.84 (-5.33, -4.35) |
| Honduras | 7 (4,10) | 0.26 (0.14,0.41) | 12 (8,18) | 0.20 (0.12,0.32) | -0.86 (-1.02, -0.70) |
| Mexico | 83 (75,100) | 0.15 (0.13,0.17) | 101 (89,112) | 0.09 (0.08,0.10) | -1.66 (-2.17, -1.15) |
| Nicaragua | 4 (2,7) | 0.14 (0.07,0.30) | 2 (1,9) | 0.05 (0.03,0.19) | -3.51 (-3.73, -3.28) |
| Panama | 3 (3,4) | 0.19 (0.16,0.21) | 3 (2,3) | 0.06 (0.05,0.07) | -3.72 (-4.07, -3.36) |
| Venezuela (Bolivarian Republic of) | 14 (12,16) | 0.11 (0.09,0.12) | 12 (9,15) | 0.04 (0.03,0.06) | -2.57 (-3.08, -2.05) |
| Brazil | 392 (371,410) | 0.38 (0.36,0.40) | 775 (710,818) | 0.33 (0.30,0.34) | -0.51 (-0.79, -0.24) |
| Paraguay | 2 (2,7) | 0.09 (0.05,0.27) | 4 (3,12) | 0.07 (0.04,0.22) | -0.54 (-0.77, -0.31) |
| Algeria | 52 (34,94) | 0.31 (0.19,0.67) | 119 (43,185) | 0.40 (0.13,0.61) | 0.93 (0.55, 1.31) |
| Bahrain | 0 (0,1) | 0.19 (0.14,0.51) | 1 (1,2) | 0.16 (0.07,0.31) | -0.72 (-1.54, 0.10) |
| Egypt | 727 (365,1033) | 1.17 (0.77,1.55) | 282 (220,377) | 0.36 (0.28,0.50) | -3.72 (-4.02, -3.41) |
| Iran (Islamic Republic of) | 471 (307,593) | 1.40 (0.89,1.94) | 429 (286,494) | 0.61 (0.40,0.70) | -2.62 (-2.76, -2.48) |
| Iraq | 37 (21,57) | 0.24 (0.13,0.49) | 38 (20,80) | 0.15 (0.08,0.38) | -1.60 (-1.73, -1.47) |
| Jordan | 2 (1,7) | 0.06 (0.03,0.29) | 4 (2,14) | 0.06 (0.02,0.20) | -0.07 (-0.42, 0.29) |
| Kuwait | 1 (1,1) | 0.07 (0.07,0.08) | 4 (3,5) | 0.14 (0.12,0.16) | 2.40 (0.11, 4.73) |
| Lebanon | 21 (13,31) | 0.89 (0.55,1.34) | 23 (17,38) | 0.37 (0.28,0.59) | -2.79 (-3.03, -2.56) |
| Libya | 15 (9,27) | 0.32 (0.20,0.64) | 18 (8,31) | 0.37 (0.17,0.64) | 0.50 (-0.24, 1.24) |
| Morocco | 72 (40,126) | 0.33 (0.18,0.67) | 131 (50,201) | 0.43 (0.15,0.67) | 0.92 (0.78, 1.06) |
| Palestine | 4 (2,8) | 0.23 (0.10,0.59) | 4 (2,11) | 0.13 (0.05,0.41) | -1.86 (-2.02, -1.69) |
| Oman | 1 (1,4) | 0.11 (0.05,0.39) | 3 (1,5) | 0.11 (0.04,0.24) | 0.14 (-0.39, 0.67) |
| Qatar | 0 (0,1) | 0.17 (0.11,0.38) | 1 (1,2) | 0.13 (0.07,0.19) | -0.90 (-1.84, 0.05) |
| Saudi Arabia | 14 (9,31) | 0.14 (0.08,0.39) | 18 (9,36) | 0.08 (0.04,0.20) | -1.79 (-2.18, -1.39) |
| Syrian Arab Republic | 10 (5,38) | 0.09 (0.05,0.43) | 6 (3,33) | 0.06 (0.03,0.29) | -1.76 (-2.14, -1.38) |
| Tunisia | 15 (10,28) | 0.25 (0.16,0.54) | 40 (12,67) | 0.36 (0.11,0.60) | 1.07 (0.83, 1.31) |
| Turkey | 538 (331,724) | 1.20 (0.79,1.65) | 408 (313,562) | 0.50 (0.39,0.69) | -2.81 (-3.00, -2.62) |
| United Arab Emirates | 6 (3,10) | 0.73 (0.38,1.09) | 10 (5,12) | 0.38 (0.20,0.53) | -2.09 (-4.39, 0.27) |
| Yemen | 44 (21,73) | 0.40 (0.16,0.81) | 106 (49,160) | 0.55 (0.21,0.90) | 0.99 (0.63, 1.34) |
| Afghanistan | 38 (19,61) | 0.41 (0.21,0.73) | 125 (56,189) | 0.67 (0.24,1.10) | 1.63 (1.47, 1.79) |
| Bangladesh | 263 (124,437) | 0.43 (0.16,0.89) | 383 (189,664) | 0.30 (0.14,0.57) | -1.00 (-1.29, -0.71) |
| Bhutan | 1 (1,2) | 0.39 (0.17,0.67) | 2 (1,3) | 0.31 (0.15,0.52) | -0.76 (-0.90, -0.63) |
| India | 1756 (1132,2495) | 0.29 (0.17,0.46) | 2612 (1739,4060) | 0.24 (0.15,0.39) | -0.63 (-1.00, -0.25) |
| Nepal | 54 (31,85) | 0.38 (0.17,0.67) | 68 (34,117) | 0.31 (0.15,0.58) | -0.66 (-0.74, -0.59) |
| Pakistan | 311 (190,458) | 0.34 (0.18,0.55) | 485 (311,747) | 0.32 (0.18,0.56) | -0.20 (-0.35, -0.06) |
| Angola | 18 (11,39) | 0.27 (0.13,0.56) | 30 (16,57) | 0.19 (0.09,0.36) | -1.09 (-1.32, -0.87) |
| Central African Republic | 5 (3,11) | 0.30 (0.14,0.65) | 8 (4,14) | 0.25 (0.10,0.45) | -0.69 (-0.77, -0.61) |
| Congo | 3 (2,7) | 0.22 (0.13,0.45) | 4 (3,8) | 0.15 (0.08,0.29) | -1.28 (-1.45, -1.11) |
| Democratic Republic of the Congo | 58 (36,105) | 0.23 (0.10,0.46) | 86 (36,161) | 0.19 (0.07,0.39) | -0.61 (-0.70, -0.52) |
| Equatorial Guinea | 1 (0,1) | 0.26 (0.12,0.55) | 1 (0,1) | 0.11 (0.06,0.20) | -2.63 (-2.92, -2.35) |
| Gabon | 1 (1,3) | 0.21 (0.10,0.45) | 1 (1,3) | 0.13 (0.07,0.24) | -1.50 (-1.55, -1.44) |
| Burundi | 16 (9,31) | 0.37 (0.15,0.83) | 16 (6,32) | 0.21 (0.07,0.44) | -1.87 (-1.98, -1.75) |
| Comoros | 1 (1,2) | 0.27 (0.12,0.50) | 1 (0,2) | 0.15 (0.07,0.30) | -1.95 (-2.72, -1.17) |
| Djibouti | 1 (0,1) | 0.23 (0.11,0.49) | 1 (1,2) | 0.15 (0.07,0.29) | -1.24 (-1.57, -0.90) |
| Eritrea | 7 (4,14) | 0.30 (0.14,0.66) | 9 (4,16) | 0.21 (0.09,0.43) | -1.14 (-1.23, -1.04) |
| Ethiopia | 92 (48,174) | 0.27 (0.10,0.54) | 97 (38,203) | 0.15 (0.05,0.30) | -1.93 (-2.01, -1.84) |
| Kenya | 19 (10,40) | 0.14 (0.05,0.32) | 33 (14,65) | 0.12 (0.05,0.23) | -0.55 (-0.69, -0.42) |
| Madagascar | 38 (23,75) | 0.42 (0.20,0.80) | 58 (31,112) | 0.31 (0.15,0.61) | -0.94 (-1.00, -0.87) |
| Malawi | 17 (10,32) | 0.22 (0.10,0.43) | 19 (9,33) | 0.16 (0.07,0.30) | -0.87 (-0.99, -0.75) |
| Mauritius | 2 (2,2) | 0.26 (0.24,0.28) | 11 (10,12) | 0.71 (0.63,0.77) | 3.71 (2.34, 5.09) |
| Mozambique | 26 (14,47) | 0.27 (0.11,0.60) | 40 (18,70) | 0.24 (0.09,0.46) | -0.39 (-0.51, -0.27) |
| Rwanda | 18 (11,39) | 0.35 (0.17,0.75) | 14 (6,30) | 0.17 (0.07,0.37) | -2.29 (-2.54, -2.03) |
| Seychelles | 0 (0,0) | 0.12 (0.07,0.36) | 0 (0,0) | 0.08 (0.04,0.25) | -1.28 (-1.40, -1.16) |
| Somalia | 20 (8,37) | 0.36 (0.11,0.75) | 34 (9,73) | 0.27 (0.06,0.58) | -0.98 (-1.08, -0.88) |
| United Republic of Tanzania | 47 (28,89) | 0.26 (0.11,0.52) | 62 (31,113) | 0.16 (0.07,0.31) | -1.51 (-1.68, -1.35) |
| Uganda | 37 (20,68) | 0.28 (0.10,0.61) | 41 (22,72) | 0.15 (0.07,0.28) | -2.03 (-2.24, -1.81) |
| Zambia | 12 (7,25) | 0.24 (0.12,0.49) | 26 (9,52) | 0.24 (0.08,0.49) | 0.11 (-0.01, 0.23) |
| Botswana | 1 (1,2) | 0.18 (0.11,0.28) | 2 (1,2) | 0.10 (0.07,0.14) | -1.78 (-2.20, -1.36) |
| Lesotho | 1 (1,2) | 0.14 (0.09,0.24) | 2 (1,3) | 0.14 (0.10,0.20) | 0.07 (-0.09, 0.23) |
| Namibia | 1 (1,2) | 0.16 (0.11,0.23) | 2 (1,3) | 0.13 (0.09,0.19) | -0.60 (-0.71, -0.49) |
| South Africa | 29 (24,37) | 0.11 (0.08,0.14) | 46 (35,55) | 0.09 (0.07,0.11) | -0.46 (-0.65, -0.28) |
| Eswatini | 1 (1,1) | 0.18 (0.12,0.29) | 1 (1,1) | 0.13 (0.09,0.18) | -1.03 (-1.13, -0.92) |
| Zimbabwe | 8 (4,14) | 0.15 (0.08,0.25) | 18 (12,27) | 0.18 (0.12,0.26) | 0.66 (0.38, 0.94) |
| Benin | 7 (4,13) | 0.21 (0.08,0.43) | 13 (7,20) | 0.15 (0.06,0.27) | -1.05 (-1.18, -0.91) |
| Burkina Faso | 20 (9,38) | 0.32 (0.09,0.67) | 36 (17,59) | 0.27 (0.09,0.52) | -0.57 (-0.71, -0.43) |
| Cameroon | 15 (8,28) | 0.24 (0.09,0.48) | 32 (19,53) | 0.17 (0.08,0.31) | -1.15 (-1.30, -1.00) |
| Cabo Verde | 1 (0,1) | 0.21 (0.06,0.48) | 1 (0,1) | 0.13 (0.05,0.24) | -1.53 (-1.81, -1.26) |
| Chad | 11 (5,24) | 0.26 (0.07,0.64) | 27 (15,49) | 0.24 (0.08,0.47) | -0.13 (-0.31, 0.05) |
| Cote d'Ivoire | 17 (10,30) | 0.24 (0.09,0.47) | 31 (16,50) | 0.18 (0.07,0.32) | -0.99 (-1.24, -0.74) |
| Gambia | 1 (1,2) | 0.23 (0.07,0.46) | 3 (1,5) | 0.19 (0.07,0.34) | -0.73 (-1.18, -0.28) |
| Ghana | 19 (11,37) | 0.22 (0.10,0.46) | 26 (14,44) | 0.13 (0.06,0.23) | -1.71 (-1.86, -1.55) |
| Guinea | 13 (7,27) | 0.26 (0.09,0.57) | 19 (9,32) | 0.21 (0.08,0.41) | -0.64 (-0.75, -0.53) |
| Guinea-Bissau | 2 (1,4) | 0.32 (0.13,0.60) | 3 (1,5) | 0.23 (0.10,0.42) | -1.05 (-1.16, -0.95) |
| Liberia | 5 (3,10) | 0.27 (0.11,0.53) | 6 (3,11) | 0.19 (0.07,0.36) | -1.21 (-1.51, -0.91) |
| Mali | 14 (8,30) | 0.23 (0.08,0.55) | 27 (13,53) | 0.18 (0.06,0.38) | -0.76 (-0.93, -0.60) |
| Mauritania | 3 (1,6) | 0.20 (0.09,0.46) | 4 (2,7) | 0.14 (0.06,0.28) | -1.15 (-1.48, -0.82) |
| Niger | 15 (7,27) | 0.28 (0.08,0.70) | 31 (11,61) | 0.23 (0.05,0.52) | -0.66 (-0.82, -0.50) |
| Nigeria | 161 (92,296) | 0.25 (0.09,0.52) | 228 (153,332) | 0.14 (0.07,0.23) | -1.79 (-1.91, -1.67) |
| Sao Tome and Principe | 0 (0,0) | 0.16 (0.06,0.37) | 0 (0,0) | 0.13 (0.05,0.24) | -0.78 (-1.16, -0.39) |
| Senegal | 13 (7,25) | 0.25 (0.09,0.49) | 18 (7,32) | 0.18 (0.06,0.33) | -1.02 (-1.20, -0.84) |
| Sierra Leone | 10 (6,18) | 0.27 (0.12,0.49) | 12 (7,21) | 0.20 (0.08,0.34) | -1.01 (-1.17, -0.85) |
| Togo | 5 (3,9) | 0.22 (0.09,0.45) | 9 (4,15) | 0.18 (0.07,0.31) | -0.70 (-0.88, -0.53) |
| American Samoa | 0 (0,0) | 0.17 (0.12,0.39) | 0 (0,0) | 0.13 (0.07,0.28) | -0.85 (-0.89, -0.82) |
| Bermuda | 1 (1,1) | 1.73 (1.49,2.01) | 1 (1,1) | 0.57 (0.48,0.69) | -3.59 (-3.87, -3.31) |
| Cook Islands | 0 (0,0) | 0.26 (0.19,0.54) | 0 (0,0) | 0.14 (0.08,0.33) | -2.03 (-2.17, -1.89) |
| Greenland | 0 (0,0) | 0.77 (0.38,0.99) | 0 (0,0) | 0.19 (0.15,0.30) | -4.36 (-4.90, -3.82) |
| Guam | 0 (0,0) | 0.15 (0.10,0.35) | 0 (0,0) | 0.06 (0.03,0.12) | -3.26 (-3.83, -2.68) |
| Monaco | 0 (0,0) | 0.18 (0.13,0.25) | 0 (0,0) | 0.15 (0.1,0.21) | -0.56 (-0.66, -0.46) |
| Nauru | 0 (0,0) | 0.31 (0.20,0.63) | 0 (0,0) | 0.37 (0.24,1.00) | 0.49 (0.39, 0.59) |
| Niue | 0 (0,0) | 0.26 (0.17,0.61) | 0 (0,0) | 0.23 (0.14,0.52) | -0.28 (-0.61, 0.05) |
| Northern Mariana Islands | 0 (0,0) | 0.13 (0.08,0.30) | 0 (0,0) | 0.11 (0.07,0.28) | -0.56 (-0.70, -0.43) |
| Palau | 0 (0,0) | 0.19 (0.11,0.42) | 0 (0,0) | 0.13 (0.06,0.30) | -1.21 (-1.29, -1.12) |
| Puerto Rico | 21 (19,23) | 0.60 (0.55,0.65) | 6 (5,7) | 0.10 (0.08,0.12) | -5.69 (-6.36, -5.02) |
| Saint Kitts and Nevis | 0 (0,0) | 0.21 (0.18,0.28) | 0 (0,0) | 0.07 (0.06,0.09) | -3.10 (-4.08, -2.11) |
| San Marino | 0 (0,0) | 0.10 (0.07,0.22) | 0 (0,0) | 0.05 (0.03,0.12) | -2.52 (-2.90, -2.12) |
| Tokelau | 0 (0,0) | 0.29 (0.18,0.61) | 0 (0,0) | 0.29 (0.18,0.73) | 0.18 (-0.00, 0.36) |
| Tuvalu | 0 (0,0) | 0.37 (0.22,0.77) | 0 (0,0) | 0.23 (0.15,0.52) | -1.58 (-1.63, -1.53) |
| United States Virgin Islands | 0 (0,0) | 0.42 (0.24,0.53) | 0 (0,0) | 0.16 (0.11,0.26) | -3.06 (-3.58, -2.54) |
| South Sudan | 15 (8,29) | 0.32 (0.14,0.68) | 17 (9,30) | 0.24 (0.09,0.44) | -0.98 (-1.15, -0.81) |
| Sudan | 72 (37,132) | 0.37 (0.20,0.70) | 124 (60,183) | 0.42 (0.18,0.61) | 0.38 (0.23, 0.52) |

Note: AAPC, average annual percentage change; CI, confidence interval; UI, uncertainty interval.

**Supplementary Table S7** Age-standardized DALYs and AAPC of PAH in 204 countries and territories, 1990-2021.

|  | DALYs | | | | |
| --- | --- | --- | --- | --- | --- |
|  | Cases in 1990 | Age-standardized rate in 1990 (per 100,000) | Cases in 2021 | Age-standardized rate in 2021 (per 100,000) | 1990–2021 AAPC (95% CI) |
| China | 149699 (115904,202546) | 16.18 (12.63,21.6) | 150941 (99583,186503) | 8.95 (6.04,11.13) | -1.98 (-2.45, -1.51) |
| Democratic People's Republic of Korea | 1492 (1014,2586) | 8.47 (5.83,14.64) | 2285 (1613,3818) | 8.04 (5.73,13.40) | -0.15 (-0.26, -0.03) |
| Taiwan (Province of China) | 405 (382,432) | 2.40 (2.28,2.55) | 1515 (1353,1649) | 4.19 (3.77,4.54) | 1.69 (0.75, 2.64) |
| Cambodia | 722 (351,1695) | 6.65 (3.49,14.12) | 777 (500,1513) | 4.92 (3.12,10.08) | -0.96 (-1.03, -0.89) |
| Indonesia | 10341 (5841,24422) | 6.00 (3.43,13.39) | 11947 (7868,24908) | 4.61 (3.03,10.44) | -0.83 (-0.94, -0.72) |
| Lao People's Democratic Republic | 479 (154,1320) | 9.96 (3.82,24.56) | 479 (256,980) | 6.65 (3.69,13.98) | -1.29 (-1.41, -1.17) |
| Malaysia | 511 (367,1189) | 3.22 (2.26,8.39) | 757 (519,1871) | 2.48 (1.66,6.63) | -0.90 (-1.65, -0.14) |
| Maldives | 55 (33,79) | 22.06 (14.86,29.92) | 34 (27,50) | 7.48 (5.98,10.28) | -3.53 (-3.70, -3.37) |
| Myanmar | 4138 (1916,10102) | 10.14 (5.16,22.81) | 3636 (2377,7104) | 6.76 (4.43,13.78) | -1.30 (-1.41, -1.20) |
| Philippines | 3227 (2173,5819) | 4.93 (3.35,10.27) | 3531 (2584,7427) | 3.28 (2.37,7.50) | -1.33 (-1.75, -0.90) |
| Sri Lanka | 2711 (1853,4476) | 17.23 (12.16,28.77) | 3523 (2180,5135) | 15.14 (9.48,21.88) | -0.29 (-0.56, -0.02) |
| Thailand | 2105 (1431,4590) | 4.14 (2.75,10.59) | 2149 (1425,6094) | 3.09 (2.13,7.50) | -0.99 (-1.33, -0.66) |
| Timor-Leste | 76 (33,194) | 8.01 (4.14,17.11) | 88 (52,174) | 6.29 (3.77,13.06) | -0.81 (-1.18, -0.44) |
| Viet Nam | 3268 (2110,6754) | 5.39 (3.27,12.30) | 3747 (2123,8856) | 3.80 (2.13,9.56) | -1.11 (-1.16, -1.07) |
| Fiji | 44 (28,78) | 7.00 (4.49,13.49) | 44 (25,83) | 5.17 (2.93,9.99) | -0.97 (-1.10, -0.83) |
| Kiribati | 7 (4,13) | 9.82 (5.55,19.6) | 8 (5,16) | 8.09 (4.85,15.83) | -0.63 (-0.69, -0.57) |
| Marshall Islands | 3 (2,6) | 10.40 (6.53,19.46) | 4 (2,7) | 8.04 (4.72,15.81) | -0.82 (-0.89, -0.76) |
| Micronesia (Federated States of) | 9 (5,16) | 10.85 (6.76,19.90) | 7 (4,13) | 7.48 (4.69,15.03) | -1.20 (-1.24, -1.16) |
| Papua New Guinea | 533 (313,852) | 12.35 (7.61,19.88) | 1243 (832,2020) | 11.31 (7.43,19.45) | -0.27 (-0.52, -0.02) |
| Samoa | 12 (8,20) | 8.64 (5.80,15.51) | 12 (8,23) | 6.56 (4.40,12.94) | -0.88 (-0.94, -0.81) |
| Solomon Islands | 21 (13,32) | 7.65 (4.64,12.89) | 34 (23,59) | 6.31 (4.18,11.93) | -0.64 (-0.90, -0.37) |
| Tonga | 5 (4,9) | 6.11 (4.36,11.58) | 4 (3,9) | 4.46 (2.97,9.33) | -1.02 (-1.13, -0.91) |
| Vanuatu | 13 (8,21) | 9.35 (5.88,16.81) | 22 (14,41) | 7.80 (5.12,15.18) | -0.55 (-0.88, -0.22) |
| Armenia | 256 (222,298) | 8.12 (7.03,9.55) | 81 (67,94) | 2.18 (1.82,2.53) | -4.10 (-4.93, -3.26) |
| Azerbaijan | 1503 (949,2126) | 22.6 (14.6,31.48) | 1871 (1078,2999) | 17.64 (10.69,27.74) | -0.84 (-1.07, -0.60) |
| Georgia | 756 (585,946) | 13.22 (10.31,16.55) | 1318 (1039,1665) | 27.83 (22.18,34.82) | 2.31 (1.71, 2.92) |
| Kazakhstan | 264 (217,310) | 1.74 (1.42,2.06) | 268 (220,316) | 1.49 (1.23,1.74) | -0.53 (-0.95, -0.10) |
| Kyrgyzstan | 138 (116,164) | 3.48 (2.92,4.17) | 105 (86,131) | 1.76 (1.46,2.16) | -2.21 (-3.14, -1.28) |
| Mongolia | 801 (407,1220) | 50.71 (26.29,79.6) | 1230 (721,1614) | 43.92 (25.60,56.54) | -0.49 (-0.85, -0.12) |
| Tajikistan | 1916 (1193,2736) | 39.71 (24.24,53.52) | 2491 (1593,3452) | 27.43 (17.75,37.43) | -1.23 (-1.38, -1.08) |
| Turkmenistan | 196 (163,245) | 6.31 (5.00,7.84) | 380 (290,499) | 7.85 (5.96,10.27) | 0.74 (0.35, 1.13) |
| Uzbekistan | 3241 (2716,3827) | 17.14 (14.13,20.56) | 3874 (3133,4699) | 12.2 (9.83,14.82) | -1.13 (-1.84, -0.42) |
| Albania | 458 (291,581) | 16.1 (10.58,20.33) | 287 (173,512) | 8.43 (5.32,14.16) | -2.10 (-2.56, -1.64) |
| Bosnia and Herzegovina | 344 (245,459) | 7.89 (5.70,10.57) | 295 (212,381) | 5.91 (4.25,7.68) | -0.92 (-1.28, -0.57) |
| Bulgaria | 485 (412,565) | 4.89 (4.14,5.68) | 286 (217,376) | 2.87 (2.19,3.74) | -1.67 (-2.35, -0.99) |
| Croatia | 191 (169,218) | 3.46 (3.07,3.92) | 92 (78,108) | 1.31 (1.11,1.53) | -3.18 (-3.90, -2.45) |
| Czechia | 1445 (1216,1725) | 12.03 (10.05,14.42) | 1899 (1608,2170) | 11.11 (9.35,12.86) | -0.27 (-1.21, 0.68) |
| Hungary | 1268 (1113,1451) | 10.13 (8.93,11.56) | 725 (590,908) | 4.66 (3.75,5.82) | -2.38 (-3.19, -1.57) |
| North Macedonia | 89 (64,133) | 4.72 (3.35,7.28) | 90 (47,153) | 3.18 (1.68,5.83) | -1.19 (-1.65, -0.73) |
| Montenegro | 10 (5,41) | 1.54 (0.85,6.81) | 8 (5,38) | 1.05 (0.60,4.82) | -1.11 (-1.50, -0.72) |
| Poland | 2435 (2143,2770) | 6.04 (5.34,6.87) | 2112 (1912,2297) | 3.95 (3.58,4.29) | -1.51 (-1.90, -1.12) |
| Romania | 3536 (2925,4196) | 14.19 (11.85,16.68) | 3949 (3384,4491) | 13.92 (12.01,15.83) | -0.12 (-0.47, 0.22) |
| Serbia | 369 (262,789) | 3.76 (2.64,8.71) | 337 (215,618) | 2.43 (1.58,4.62) | -1.37 (-1.56, -1.17) |
| Slovakia | 190 (129,326) | 3.40 (2.31,6.14) | 154 (100,268) | 2.04 (1.32,3.61) | -1.68 (-1.91, -1.46) |
| Slovenia | 30 (26,34) | 1.29 (1.13,1.48) | 38 (33,44) | 1.03 (0.88,1.21) | -0.86 (-1.40, -0.31) |
| Belarus | 222 (185,282) | 2.22 (1.86,2.94) | 185 (154,220) | 1.63 (1.37,1.89) | -1.16 (-1.34, -0.97) |
| Estonia | 19 (16,22) | 1.17 (1.02,1.36) | 24 (21,28) | 1.12 (0.96,1.29) | -0.27 (-2.41, 1.92) |
| Latvia | 33 (28,39) | 1.17 (1.02,1.36) | 89 (77,102) | 2.89 (2.51,3.30) | 2.57 (1.22, 3.94) |
| Lithuania | 63 (55,72) | 1.71 (1.51,1.95) | 100 (87,113) | 2.30 (2.04,2.58) | 0.70 (-1.15, 2.57) |
| Republic of Moldova | 17 (13,23) | 0.39 (0.29,0.52) | 22 (18,29) | 0.54 (0.44,0.68) | 0.95 (0.10, 1.81) |
| Russian Federation | 18577 (16747,21771) | 12.67 (11.52,14.59) | 6389 (5934,6897) | 3.62 (3.38,3.89) | -3.97 (-4.89, -3.03) |
| Ukraine | 1697 (1533,1901) | 2.99 (2.69,3.39) | 1547 (1172,1956) | 3.05 (2.37,3.83) | 0.05 (-1.52, 1.66) |
| Brunei Darussalam | 54 (28,72) | 20.62 (11.28,27.62) | 45 (27,58) | 11.44 (7.20,14.88) | -1.93 (-2.22, -1.64) |
| Japan | 15634 (15119,16190) | 14.12 (13.66,14.6) | 18490 (16064,20370) | 10.72 (10.03,11.46) | -0.89 (-1.43, -0.35) |
| Republic of Korea | 2475 (1692,3462) | 6.52 (4.49,9.50) | 1246 (842,2906) | 2.40 (1.67,4.75) | -3.08 (-3.42, -2.74) |
| Singapore | 311 (289,335) | 11.63 (10.81,12.49) | 207 (187,228) | 3.83 (3.44,4.27) | -3.82 (-4.89, -2.72) |
| Australia | 1322 (1151,1672) | 7.82 (6.77,9.87) | 1207 (1087,1333) | 3.69 (3.36,4.03) | -2.36 (-2.73, -1.98) |
| New Zealand | 192 (178,206) | 5.33 (4.94,5.71) | 226 (207,249) | 3.55 (3.26,3.89) | -1.18 (-2.24, -0.11) |
| Andorra | 4 (3,6) | 9.24 (6.48,12.64) | 4 (3,6) | 3.55 (2.51,4.90) | -3.13 (-3.50, -2.76) |
| Austria | 509 (475,545) | 5.45 (5.12,5.82) | 495 (450,542) | 3.47 (3.21,3.77) | -1.43 (-1.72, -1.13) |
| Belgium | 1112 (991,1389) | 9.49 (8.41,11.94) | 900 (801,981) | 5.18 (4.74,5.60) | -2.13 (-2.25, -2.00) |
| Cyprus | 377 (205,488) | 52.32 (28.85,66.39) | 305 (166,379) | 17.45 (9.61,21.85) | -3.56 (-4.06, -3.06) |
| Denmark | 474 (438,513) | 8.64 (7.98,9.36) | 426 (384,462) | 4.80 (4.39,5.20) | -1.83 (-2.24, -1.42) |
| Finland | 173 (152,195) | 3.12 (2.74,3.50) | 178 (160,198) | 2.13 (1.95,2.35) | -1.20 (-1.56, -0.85) |
| France | 5949 (4979,7308) | 9.04 (7.70,11.20) | 6113 (5521,6689) | 6.05 (5.60,6.55) | -1.27 (-1.63, -0.90) |
| Germany | 8412 (7108,9616) | 9.48 (8.04,10.81) | 9441 (8306,10352) | 6.42 (5.86,6.91) | -1.29 (-1.81, -0.77) |
| Greece | 860 (809,912) | 7.86 (7.37,8.31) | 1495 (1340,1645) | 8.35 (7.67,9.13) | 0.14 (-0.46, 0.75) |
| Iceland | 14 (13,16) | 5.55 (5.16,5.99) | 17 (15,19) | 3.79 (3.36,4.27) | -1.19 (-1.67, -0.71) |
| Ireland | 168 (158,180) | 4.59 (4.34,4.90) | 190 (172,209) | 3.02 (2.74,3.33) | -1.37 (-1.85, -0.89) |
| Israel | 797 (706,1034) | 16.23 (14.4,21.01) | 796 (719,869) | 7.18 (6.52,7.81) | -2.65 (-2.84, -2.46) |
| Italy | 4731 (4493,4968) | 7.16 (6.83,7.49) | 2779 (2504,3033) | 2.78 (2.58,2.99) | -3.09 (-3.46, -2.72) |
| Luxembourg | 36 (33,38) | 8.29 (7.72,8.79) | 46 (41,51) | 5.06 (4.54,5.64) | -1.63 (-2.06, -1.21) |
| Malta | 12 (11,13) | 3.19 (2.93,3.48) | 18 (16,20) | 2.90 (2.55,3.27) | -0.28 (-0.71, 0.14) |
| Netherlands | 873 (819,938) | 5.38 (5.07,5.74) | 1004 (915,1106) | 3.91 (3.61,4.26) | -1.04 (-1.35, -0.73) |
| Norway | 287 (274,301) | 7.21 (6.89,7.52) | 133 (121,148) | 1.92 (1.77,2.11) | -4.55 (-6.55, -2.50) |
| Portugal | 886 (833,941) | 8.98 (8.47,9.56) | 1022 (917,1117) | 5.82 (5.35,6.28) | -1.48 (-2.11, -0.85) |
| Spain | 3743 (3526,3949) | 9.18 (8.66,9.70) | 4292 (3845,4655) | 6.00 (5.52,6.41) | -1.42 (-1.78, -1.06) |
| Sweden | 578 (541,617) | 6.08 (5.70,6.46) | 604 (540,678) | 4.14 (3.73,4.63) | -1.29 (-2.21, -0.36) |
| Switzerland | 927 (811,1136) | 12.09 (10.29,14.95) | 715 (634,794) | 5.12 (4.64,5.63) | -2.72 (-2.97, -2.47) |
| United Kingdom | 3646 (3234,4483) | 6.44 (5.57,7.93) | 3037 (2872,3181) | 3.73 (3.54,3.92) | -1.56 (-1.74, -1.38) |
| Argentina | 6519 (5775,7312) | 19.89 (17.6,22.29) | 3409 (3183,3695) | 7.12 (6.62,7.76) | -3.28 (-3.89, -2.66) |
| Chile | 1159 (1084,1238) | 9.04 (8.49,9.64) | 1112 (1028,1201) | 5.45 (4.99,5.90) | -1.63 (-1.88, -1.37) |
| Uruguay | 305 (273,337) | 9.71 (8.69,10.74) | 218 (200,238) | 5.44 (4.97,5.99) | -1.97 (-2.26, -1.68) |
| Canada | 3066 (2872,3264) | 11.37 (10.64,12.11) | 2171 (1982,2337) | 4.63 (4.23,4.99) | -2.64 (-3.14, -2.13) |
| United States of America | 27123 (24675,29590) | 10.00 (9.2,10.94) | 36198 (33004,38648) | 8.08 (7.49,8.60) | -0.69 (-0.86, -0.51) |
| Antigua and Barbuda | 3 (2,3) | 4.42 (3.93,5.05) | 1 (1,2) | 1.50 (1.37,1.64) | -3.42 (-3.89, -2.94) |
| Bahamas | 105 (93,119) | 44.29 (39.24,49.69) | 59 (47,75) | 14.75 (11.70,18.62) | -3.50 (-3.86, -3.14) |
| Barbados | 97 (84,110) | 37.98 (32.89,42.51) | 43 (34,54) | 11.88 (9.32,15.07) | -3.45 (-3.94, -2.95) |
| Belize | 51 (45,58) | 23.42 (21.30,26.33) | 22 (20,25) | 5.72 (5.03,6.45) | -4.46 (-4.84, -4.07) |
| Cuba | 484 (445,534) | 4.69 (4.31,5.19) | 265 (228,301) | 1.90 (1.64,2.14) | -2.66 (-2.97, -2.35) |
| Dominica | 4 (3,6) | 6.07 (3.90,8.16) | 2 (2,4) | 3.46 (2.49,5.66) | -1.80 (-1.94, -1.65) |
| Dominican Republic | 842 (528,1156) | 9.71 (7.02,13.14) | 456 (330,832) | 4.26 (3.05,7.94) | -2.58 (-2.75, -2.41) |
| Grenada | 28 (24,33) | 32.89 (28.29,38.70) | 9 (8,11) | 8.80 (7.66,10.03) | -4.16 (-4.50, -3.82) |
| Guyana | 79 (68,92) | 8.66 (7.69,9.79) | 119 (91,157) | 16.06 (12.29,21.11) | 2.15 (1.51, 2.79) |
| Haiti | 3923 (1303,7297) | 45.54 (17.74,78.68) | 3454 (1446,6073) | 25.17 (11.87,42.29) | -1.85 (-2.01, -1.70) |
| Jamaica | 230 (200,265) | 9.67 (8.40,11.00) | 78 (60,100) | 2.81 (2.16,3.62) | -3.86 (-4.42, -3.30) |
| Saint Lucia | 35 (30,39) | 28.45 (25.20,31.93) | 16 (13,19) | 8.36 (6.78,10.22) | -3.89 (-4.51, -3.28) |
| Saint Vincent and the Grenadines | 5 (4,5) | 4.30 (3.81,4.87) | 3 (2,3) | 2.45 (2.08,2.88) | -1.81 (-2.29, -1.33) |
| Suriname | 143 (78,178) | 36.76 (21.37,45.17) | 79 (58,109) | 14.26 (10.62,19.62) | -3.02 (-3.40, -2.64) |
| Trinidad and Tobago | 233 (209,260) | 20.94 (18.88,23.27) | 110 (85,142) | 7.72 (5.91,9.91) | -3.17 (-3.71, -2.64) |
| Bolivia (Plurinational State of) | 1591 (718,2702) | 19.49 (10.24,31.46) | 980 (708,1392) | 9.02 (6.57,12.82) | -2.45 (-2.49, -2.40) |
| Ecuador | 954 (802,1234) | 9.56 (7.77,11.99) | 957 (812,1116) | 5.80 (4.94,6.74) | -1.71 (-2.07, -1.35) |
| Peru | 2504 (1458,3769) | 10.57 (6.89,14.85) | 1607 (1197,2184) | 4.65 (3.45,6.29) | -2.49 (-3.34, -1.64) |
| Colombia | 1787 (1488,2250) | 5.81 (4.85,7.26) | 1645 (1380,1949) | 3.29 (2.74,3.93) | -1.85 (-2.17, -1.54) |
| Costa Rica | 311 (289,335) | 12.40 (11.51,13.34) | 164 (145,183) | 3.31 (2.92,3.70) | -4.49 (-5.02, -3.96) |
| El Salvador | 677 (422,882) | 11.91 (7.96,15.04) | 263 (190,441) | 4.18 (3.02,6.98) | -3.30 (-3.46, -3.15) |
| Guatemala | 863 (757,1064) | 9.37 (8.31,11.15) | 324 (275,386) | 2.36 (2.02,2.80) | -4.50 (-4.88, -4.13) |
| Honduras | 338 (215,467) | 8.15 (5.15,11.98) | 409 (283,617) | 5.30 (3.67,8.02) | -1.34 (-1.53, -1.16) |
| Mexico | 4819 (4095,5871) | 5.59 (5.00,6.65) | 3759 (3349,4226) | 3.11 (2.78,3.53) | -1.89 (-2.39, -1.40) |
| Nicaragua | 279 (143,397) | 6.13 (3.44,9.41) | 102 (69,302) | 1.76 (1.18,5.35) | -3.97 (-4.13, -3.81) |
| Panama | 132 (114,150) | 6.27 (5.44,7.04) | 89 (74,104) | 2.08 (1.73,2.46) | -3.47 (-3.79, -3.15) |
| Venezuela (Bolivarian Republic of) | 759 (657,866) | 4.25 (3.69,4.81) | 492 (391,609) | 1.83 (1.46,2.27) | -2.53 (-3.70, -1.35) |
| Brazil | 18943 (17689,20254) | 14.51 (13.64,15.35) | 24078 (22850,25249) | 10.45 (9.88,11.02) | -1.06 (-1.42, -0.70) |
| Paraguay | 122 (87,254) | 3.18 (2.24,7.27) | 157 (103,384) | 2.42 (1.58,6.14) | -0.87 (-1.21, -0.53) |
| Algeria | 3350 (2116,5773) | 12.33 (8.00,22.02) | 4335 (2024,6496) | 11.09 (4.89,16.62) | -0.30 (-0.49, -0.12) |
| Bahrain | 29 (21,56) | 6.60 (4.94,14.25) | 48 (24,83) | 4.52 (2.26,8.02) | -1.29 (-2.07, -0.50) |
| Egypt | 58615 (23940,86262) | 74.21 (37.61,105.38) | 17676 (13635,23381) | 16.51 (12.82,21.87) | -4.77 (-5.10, -4.44) |
| Iran (Islamic Republic of) | 28135 (17155,38911) | 50.93 (32.60,63.56) | 12801 (9989,14672) | 16.26 (12.86,18.66) | -3.60 (-3.83, -3.37) |
| Iraq | 2476 (1291,3562) | 11.13 (6.20,16.49) | 1822 (993,3158) | 5.20 (2.84,10.09) | -2.46 (-2.65, -2.28) |
| Jordan | 122 (67,444) | 2.77 (1.49,10.88) | 253 (124,686) | 2.45 (1.20,6.89) | -0.39 (-0.74, -0.04) |
| Kuwait | 68 (61,78) | 4.17 (3.73,4.70) | 207 (174,243) | 6.51 (5.41,7.73) | 1.49 (-1.08, 4.13) |
| Lebanon | 948 (529,1451) | 31.97 (18.96,48.19) | 617 (485,814) | 11.26 (8.86,14.49) | -3.38 (-3.66, -3.10) |
| Libya | 1146 (647,2165) | 20.28 (11.95,37.85) | 1004 (437,1782) | 20.43 (8.75,36.65) | -0.00 (-0.76, 0.76) |
| Morocco | 4665 (2537,7927) | 15.60 (8.74,26.69) | 4559 (2226,6953) | 13.46 (6.51,20.47) | -0.48 (-0.66, -0.30) |
| Palestine | 293 (131,482) | 10.15 (4.91,19.39) | 212 (91,500) | 4.66 (1.98,11.88) | -2.50 (-2.73, -2.27) |
| Oman | 87 (46,226) | 4.18 (2.22,12.07) | 134 (51,241) | 3.90 (1.49,7.22) | -0.22 (-0.67, 0.23) |
| Qatar | 19 (13,37) | 5.34 (3.73,10.72) | 69 (37,102) | 3.75 (1.96,5.57) | -1.21 (-1.95, -0.46) |
| Saudi Arabia | 952 (573,1681) | 5.65 (3.56,11.82) | 872 (465,1518) | 2.62 (1.45,5.39) | -2.50 (-2.88, -2.12) |
| Syrian Arab Republic | 732 (373,2390) | 4.54 (2.50,16.61) | 284 (133,1390) | 2.32 (1.05,11.57) | -2.17 (-2.49, -1.85) |
| Tunisia | 901 (574,1529) | 10.56 (6.84,18.90) | 1064 (438,1721) | 9.23 (3.88,14.80) | -0.46 (-0.64, -0.27) |
| Turkey | 31949 (16439,49530) | 53.91 (30.72,77.55) | 12652 (9856,15875) | 16.56 (12.93,20.56) | -3.76 (-3.89, -3.63) |
| United Arab Emirates | 418 (232,632) | 26.79 (14.5,38.89) | 434 (266,541) | 10.31 (5.63,13.04) | -3.06 (-4.19, -1.92) |
| Yemen | 3171 (1563,5313) | 16.75 (8.15,27.97) | 5975 (2917,8721) | 19.30 (9.07,28.92) | 0.46 (0.20, 0.72) |
| Afghanistan | 2360 (1186,3637) | 18.19 (9.20,29.19) | 8110 (3768,12131) | 25.28 (10.98,39.03) | 1.11 (0.89, 1.32) |
| Bangladesh | 13289 (7185,19200) | 13.28 (6.39,21.85) | 12634 (7167,19514) | 8.66 (4.81,13.69) | -1.32 (-1.51, -1.14) |
| Bhutan | 92 (45,137) | 14.29 (7.77,22.41) | 61 (34,97) | 9.27 (5.13,14.22) | -1.36 (-1.71, -1.02) |
| India | 99575 (57534,134279) | 11.73 (7.49,16.30) | 94737 (69017,131902) | 7.75 (5.52,11.36) | -1.32 (-1.52, -1.11) |
| Nepal | 3424 (1746,5125) | 14.75 (8.37,23.03) | 2557 (1465,3967) | 9.42 (5.15,15.02) | -1.43 (-1.55, -1.32) |
| Pakistan | 19706 (10809,28216) | 14.41 (8.92,20.63) | 26574 (16921,38115) | 11.96 (7.86,18.26) | -0.61 (-0.82, -0.40) |
| Angola | 1120 (597,2655) | 10.14 (6.07,21.29) | 1628 (976,2924) | 6.21 (3.27,11.56) | -1.55 (-1.79, -1.32) |
| Central African Republic | 324 (177,698) | 11.36 (6.34,21.84) | 429 (232,780) | 8.92 (4.34,15.95) | -0.82 (-0.91, -0.73) |
| Congo | 173 (114,372) | 7.91 (5.31,16.16) | 206 (131,369) | 4.80 (2.93,8.78) | -1.56 (-1.80, -1.31) |
| Democratic Republic of the Congo | 3648 (2260,6621) | 8.75 (5.31,15.90) | 4158 (2013,7024) | 6.13 (2.66,11.34) | -1.14 (-1.28, -0.99) |
| Equatorial Guinea | 41 (22,83) | 9.48 (5.49,17.84) | 43 (24,75) | 3.83 (2.13,6.65) | -2.89 (-3.22, -2.55) |
| Gabon | 61 (39,108) | 6.99 (4.07,13.04) | 60 (34,104) | 4.14 (2.32,7.11) | -1.67 (-1.92, -1.41) |
| Burundi | 981 (592,1971) | 15.6 (8.34,32.70) | 916 (414,1813) | 7.64 (3.08,15.11) | -2.31 (-2.54, -2.08) |
| Comoros | 62 (35,108) | 11.73 (6.64,19.72) | 41 (23,81) | 5.92 (3.15,11.42) | -2.30 (-3.44, -1.14) |
| Djibouti | 39 (20,78) | 9.30 (5.13,18.30) | 63 (30,119) | 5.68 (2.66,10.81) | -1.49 (-1.92, -1.05) |
| Eritrea | 460 (256,967) | 13.09 (7.07,27.04) | 470 (239,931) | 8.01 (3.8,15.23) | -1.67 (-1.74, -1.61) |
| Ethiopia | 5663 (3177,11304) | 10.79 (5.32,20.84) | 5398 (2500,11569) | 5.53 (2.30,11.22) | -2.15 (-2.29, -2.02) |
| Kenya | 1164 (748,2248) | 5.22 (2.64,10.69) | 1655 (820,3204) | 4.05 (1.93,7.82) | -0.82 (-0.91, -0.73) |
| Madagascar | 2391 (1395,5242) | 18.04 (10.75,35.43) | 3454 (1961,6745) | 12.80 (6.85,24.47) | -1.11 (-1.17, -1.05) |
| Malawi | 1165 (640,2319) | 9.49 (5.69,17.02) | 1046 (546,1827) | 6.17 (3.08,10.65) | -1.32 (-1.60, -1.04) |
| Mauritius | 110 (102,119) | 10.91 (10.11,11.82) | 398 (351,434) | 28.96 (25.61,31.57) | 3.75 (1.92, 5.61) |
| Mozambique | 1573 (837,2789) | 10.78 (5.94,20.22) | 2157 (1087,3608) | 8.59 (3.81,15.15) | -0.75 (-0.95, -0.56) |
| Rwanda | 1150 (686,2622) | 14.89 (8.47,30.80) | 725 (356,1529) | 6.17 (2.87,13.12) | -2.75 (-3.27, -2.22) |
| Seychelles | 3 (2,6) | 4.26 (2.88,9.42) | 3 (2,7) | 2.86 (1.75,6.44) | -0.95 (-1.61, -0.28) |
| Somalia | 1328 (604,2412) | 15.22 (5.96,29.72) | 2163 (667,4513) | 10.58 (2.79,22.97) | -1.15 (-1.35, -0.95) |
| United Republic of Tanzania | 2892 (1921,5624) | 10.23 (5.60,18.80) | 3514 (2012,6433) | 6.27 (3.28,11.36) | -1.58 (-1.76, -1.40) |
| Uganda | 2518 (1509,4482) | 11.72 (5.79,22.80) | 2531 (1529,4433) | 5.98 (3.20,10.36) | -2.16 (-2.55, -1.78) |
| Zambia | 771 (450,1777) | 9.20 (5.51,18.24) | 1343 (567,2581) | 8.64 (3.22,17.19) | -0.18 (-0.34, -0.02) |
| Botswana | 69 (46,113) | 6.58 (4.32,10.65) | 86 (60,114) | 4.04 (2.86,5.30) | -1.62 (-2.04, -1.20) |
| Lesotho | 62 (39,105) | 4.93 (3.06,8.21) | 87 (59,116) | 5.40 (3.74,7.19) | 0.29 (0.13, 0.46) |
| Namibia | 66 (47,98) | 5.88 (4.21,8.48) | 99 (67,141) | 4.86 (3.36,6.76) | -0.60 (-0.73, -0.47) |
| South Africa | 1545 (1220,1881) | 4.50 (3.66,5.65) | 1932 (1491,2297) | 3.58 (2.77,4.23) | -0.73 (-1.08, -0.38) |
| Eswatini | 44 (31,68) | 6.76 (4.70,10.53) | 53 (35,73) | 5.30 (3.57,7.29) | -0.76 (-0.95, -0.57) |
| Zimbabwe | 402 (207,678) | 5.22 (2.65,8.94) | 955 (595,1432) | 7.16 (4.65,10.44) | 1.11 (0.65, 1.56) |
| Benin | 442 (271,851) | 7.98 (4.18,14.04) | 780 (487,1192) | 5.73 (2.98,9.04) | -1.05 (-1.21, -0.89) |
| Burkina Faso | 1142 (637,1963) | 11.14 (4.83,21.45) | 1992 (1162,3181) | 8.98 (4.20,14.92) | -0.68 (-0.84, -0.52) |
| Cameroon | 846 (539,1528) | 8.38 (4.19,15.26) | 1814 (1238,2862) | 6.27 (3.78,10.57) | -0.92 (-1.04, -0.81) |
| Cabo Verde | 33 (16,64) | 8.63 (3.53,18.25) | 22 (9,41) | 4.41 (1.75,8.30) | -2.13 (-2.35, -1.92) |
| Chad | 664 (310,1316) | 9.49 (4.12,20.39) | 1766 (980,3156) | 9.18 (4.46,16.36) | -0.05 (-0.19, 0.09) |
| Cote d'Ivoire | 1114 (729,1989) | 8.96 (4.77,15.04) | 1818 (1114,2742) | 6.79 (3.56,10.99) | -0.89 (-1.13, -0.65) |
| Gambia | 79 (46,145) | 8.36 (3.72,15.32) | 134 (70,241) | 6.52 (3.06,11.69) | -0.79 (-1.73, 0.16) |
| Ghana | 1066 (720,2056) | 8.01 (4.51,14.84) | 1256 (806,2062) | 4.49 (2.65,7.35) | -1.85 (-1.97, -1.74) |
| Guinea | 773 (421,1603) | 10.26 (5.42,21.05) | 1090 (642,1803) | 8.02 (3.99,13.76) | -0.78 (-0.97, -0.60) |
| Guinea-Bissau | 126 (74,252) | 12.24 (6.65,22.93) | 153 (85,261) | 8.52 (4.19,14.99) | -1.08 (-1.18, -0.98) |
| Liberia | 354 (202,711) | 11.46 (6.85,20.21) | 339 (173,575) | 6.94 (3.10,12.33) | -1.76 (-2.19, -1.32) |
| Mali | 879 (465,1797) | 9.00 (4.50,19.24) | 1667 (918,2938) | 6.95 (3.08,13.92) | -0.81 (-1.07, -0.56) |
| Mauritania | 136 (86,268) | 6.92 (3.84,14.05) | 177 (101,300) | 4.73 (2.40,8.52) | -1.26 (-1.40, -1.13) |
| Niger | 955 (461,1825) | 10.39 (4.50,20.95) | 1835 (768,3526) | 7.69 (2.46,15.66) | -0.96 (-1.20, -0.72) |
| Nigeria | 9447 (6712,19379) | 9.36 (5.30,16.75) | 15081 (10537,21578) | 6.04 (4.07,8.84) | -1.38 (-1.46, -1.31) |
| Sao Tome and Principe | 9 (5,18) | 6.57 (3.75,12.34) | 8 (3,15) | 4.36 (1.85,8.40) | -1.30 (-1.55, -1.06) |
| Senegal | 820 (535,1575) | 9.81 (5.09,17.6) | 904 (433,1528) | 6.40 (2.74,11.21) | -1.26 (-1.55, -0.98) |
| Sierra Leone | 644 (353,1346) | 11.59 (6.90,19.85) | 774 (457,1268) | 8.24 (4.47,13.62) | -1.12 (-1.56, -0.69) |
| Togo | 305 (195,570) | 8.38 (4.43,15.55) | 460 (237,734) | 6.36 (3.03,10.38) | -0.91 (-1.17, -0.66) |
| American Samoa | 2 (1,3) | 5.13 (3.63,10.00) | 2 (1,4) | 4.15 (2.45,8.06) | -0.59 (-0.62, -0.55) |
| Bermuda | 34 (30,39) | 55.52 (48.52,64.73) | 16 (13,19) | 15.69 (13.06,19.07) | -3.99 (-4.70, -3.28) |
| Cook Islands | 2 (1,3) | 9.83 (7.25,17.61) | 1 (1,2) | 5.26 (3.14,10.93) | -1.86 (-2.07, -1.66) |
| Greenland | 16 (6,23) | 31.58 (13.61,44.07) | 4 (3,6) | 7.06 (5.05,10.16) | -4.72 (-5.00, -4.44) |
| Guam | 5 (4,10) | 4.52 (3.26,8.95) | 4 (2,8) | 2.53 (1.37,4.91) | -1.86 (-2.24, -1.47) |
| Monaco | 3 (2,3) | 5.54 (4.21,7.60) | 3 (2,4) | 4.46 (3.07,6.12) | -0.65 (-0.79, -0.51) |
| Nauru | 1 (1,2) | 10.68 (6.85,20.99) | 1 (1,2) | 10.30 (6.33,22.74) | -0.11 (-0.21, -0.02) |
| Niue | 0 (0,0) | 8.31 (5.42,16.73) | 0 (0,0) | 11.14 (7.04,23.27) | 1.12 (0.56, 1.68) |
| Northern Mariana Islands | 1 (1,3) | 3.90 (2.39,7.66) | 2 (1,3) | 3.36 (2.06,6.99) | -0.49 (-0.63, -0.34) |
| Palau | 1 (0,2) | 6.30 (3.58,12.73) | 1 (0,2) | 4.37 (2.21,9.17) | -1.19 (-1.30, -1.08) |
| Puerto Rico | 832 (773,899) | 23.73 (22.03,25.57) | 158 (132,185) | 3.98 (3.33,4.67) | -5.50 (-6.31, -4.69) |
| Saint Kitts and Nevis | 3 (3,4) | 8.12 (7.10,11.40) | 1 (1,2) | 2.32 (1.90,2.81) | -3.84 (-4.40, -3.27) |
| San Marino | 1 (1,2) | 3.20 (2.46,5.88) | 1 (1,2) | 1.61 (1.07,3.13) | -2.29 (-2.51, -2.06) |
| Tokelau | 0 (0,0) | 9.27 (5.82,17.72) | 0 (0,0) | 13.26 (8.10,31.56) | 1.46 (0.55, 2.38) |
| Tuvalu | 1 (1,2) | 12.52 (7.35,23.75) | 1 (1,2) | 7.30 (4.76,14.38) | -1.71 (-1.82, -1.60) |
| United States Virgin Islands | 18 (10,22) | 16.97 (9.78,21.31) | 6 (4,9) | 6.40 (4.53,8.82) | -3.00 (-3.29, -2.72) |
| South Sudan | 974 (440,1977) | 13.88 (7.57,26.60) | 1106 (628,2080) | 10.34 (5.26,18.60) | -0.93 (-1.24, -0.62) |
| Sudan | 5213 (2387,10407) | 18.24 (9.41,32.75) | 7551 (3740,11346) | 17.35 (8.60,25.58) | -0.16 (-0.24, -0.08) |

Note: AAPC, average annual percentage change; CI, confidence interval; UI, uncertainty interval; DALYs, disability-adjusted life years

**Supplementary Table S8** Predicted global age-standardized prevalence, mortality and DALYs of PAH from 2022 to 2050.

| Year | Prevalence (per 100,000) | Mortality (per 100,000) | DALYs (per 100,000) |
| --- | --- | --- | --- |
| 1990 | 2.30 | 0.35 | 13.21 |
| 1991 | 2.30 | 0.35 | 13.07 |
| 1992 | 2.30 | 0.35 | 12.94 |
| 1993 | 2.30 | 0.35 | 12.83 |
| 1994 | 2.30 | 0.35 | 12.64 |
| 1995 | 2.29 | 0.35 | 12.50 |
| 1996 | 2.29 | 0.34 | 12.24 |
| 1997 | 2.29 | 0.34 | 12.01 |
| 1998 | 2.28 | 0.33 | 11.83 |
| 1999 | 2.28 | 0.33 | 11.74 |
| 2000 | 2.28 | 0.33 | 11.59 |
| 2001 | 2.28 | 0.33 | 11.43 |
| 2002 | 2.29 | 0.33 | 11.29 |
| 2003 | 2.30 | 0.33 | 11.14 |
| 2004 | 2.31 | 0.32 | 10.93 |
| 2005 | 2.31 | 0.32 | 10.83 |
| 2006 | 2.32 | 0.32 | 10.73 |
| 2007 | 2.32 | 0.32 | 10.73 |
| 2008 | 2.32 | 0.33 | 10.81 |
| 2009 | 2.32 | 0.34 | 10.88 |
| 2010 | 2.32 | 0.34 | 10.88 |
| 2011 | 2.32 | 0.34 | 10.75 |
| 2012 | 2.32 | 0.34 | 10.57 |
| 2013 | 2.32 | 0.33 | 10.38 |
| 2014 | 2.32 | 0.32 | 10.09 |
| 2015 | 2.32 | 0.32 | 9.81 |
| 2016 | 2.32 | 0.31 | 9.53 |
| 2017 | 2.32 | 0.30 | 9.23 |
| 2018 | 2.31 | 0.29 | 8.96 |
| 2019 | 2.31 | 0.29 | 8.75 |
| 2020 | 2.29 | 0.28 | 8.42 |
| 2021 | 2.28 | 0.27 | 8.24 |
| 2022 | 2.26 | 0.27 | 8.06 |
| 2023 | 2.25 | 0.27 | 7.89 |
| 2024 | 2.24 | 0.27 | 7.72 |
| 2025 | 2.23 | 0.27 | 7.56 |
| 2026 | 2.22 | 0.27 | 7.39 |
| 2027 | 2.22 | 0.27 | 7.23 |
| 2028 | 2.22 | 0.27 | 7.07 |
| 2029 | 2.21 | 0.27 | 6.90 |
| 2030 | 2.21 | 0.27 | 6.74 |
| 2031 | 2.21 | 0.27 | 6.58 |
| 2032 | 2.21 | 0.27 | 6.42 |
| 2033 | 2.21 | 0.27 | 6.26 |
| 2034 | 2.20 | 0.27 | 6.10 |
| 2035 | 2.20 | 0.27 | 5.94 |
| 2036 | 2.20 | 0.27 | 5.78 |
| 2037 | 2.20 | 0.27 | 5.62 |
| 2038 | 2.20 | 0.27 | 5.46 |
| 2039 | 2.20 | 0.27 | 5.30 |
| 2040 | 2.20 | 0.27 | 5.14 |
| 2041 | 2.20 | 0.27 | 4.98 |
| 2042 | 2.20 | 0.27 | 4.82 |
| 2043 | 2.20 | 0.27 | 4.65 |
| 2044 | 2.20 | 0.27 | 4.49 |
| 2045 | 2.20 | 0.27 | 4.33 |
| 2046 | 2.20 | 0.27 | 4.17 |
| 2047 | 2.20 | 0.27 | 4.01 |
| 2048 | 2.20 | 0.27 | 3.85 |
| 2049 | 2.20 | 0.27 | 3.69 |
| 2050 | 2.20 | 0.27 | 3.53 |

Note: DALYs, disability-adjusted life years.
